# Across intra-mammalian stages of the liver f luke *Fasciola hepatica*: a proteomic study

**DOI:** 10.1038/srep32796

**Published:** 2016-09-07

**Authors:** Lucía Sánchez Di Maggio, Lucas Tirloni, Antonio F. M. Pinto, Jolene K. Diedrich, John R. Yates III, Uruguaysito Benavides, Carlos Carmona, Itabajara da Silva Vaz Jr., Patricia Berasain

**Affiliations:** 1 Unidad de Biología Parasitaria, Facultad de Ciencias, Universidad de la República Oriental del Uruguay, Montevideo, Uruguay; 2 Centro de Biotecnologia, Universidade Federal do Rio Grande do Sul, Porto Alegre, RS, Brazil; 3 Faculdade de Veterinária, Universidade Federal do Rio Grande do Sul, Porto Alegre, RS, Brazil; 4 Centro de Pesquisas em Biologia Molecular e Funcional, Instituto Nacional de Ciência e Tecnologia em Tuberculose, Pontifícia Universidade Católica do Rio Grande do Sul, Porto Alegre, RS, Brazil; 5 Department of Chemical Physiology, The Scripps Research Institute, CA, Unites States of America; 6 Departamento de Inmunología, Facultad de Veterinaria, Universidad de la República Oriental del Uruguay, Montevideo, Uruguay

## Abstract

*Fasciola hepatica* is the agent of fasciolosis, a foodborne zoonosis that affects livestock production and human health. Although flukicidal drugs are available, re-infection and expanding resistance to triclabendazole demand new control strategies. Understanding the molecular mechanisms underlying the complex interaction with the mammalian host could provide relevant clues, aiding the search for novel targets in diagnosis and control of fasciolosis. Parasite survival in the mammalian host is mediated by parasite compounds released during infection, known as excretory/secretory (E/S) products. E/S products are thought to protect parasites from host responses, allowing them to survive for a long period in the vertebrate host. This work provides in-depth proteomic analysis of *F. hepatica* intra-mammalian stages, and represents the largest number of proteins identified to date for this species. Functional classification revealed the presence of proteins involved in different biological processes, many of which represent original findings for this organism and are important for parasite survival within the host. These results could lead to a better comprehension of host-parasite relationships, and contribute to the development of drugs or vaccines against this parasite.

Fasciolosis is a zoonotic foodborne disease caused mostly by the digenean trematode parasites *Fasciola hepatica* and *Fasciola gigantica. F. hepatica* has a worldwide distribution, while *F. gigantica* is found in tropical climates, with a much more focal distribution in parts of Africa and Asia, where these species overlap^1^. The disease causes significant economic losses in livestock production worldwide, also having increased relevance to human health in developing countries^1^.

Current control relies mainly on the use of anthelmintic drugs, eradication of the intermediate host with molluscicides, as well as improving drainage systems to limit snails habitat^2^. Nevertheless, emerging resistance to anthelminthic drugs and the presence of xenobiotic residues in food and environment have stimulated the search for novel control methods. Immune control through the development of vaccines has emerged as a promising alternative; however, vaccines have to reach an appropriate level of efficacy to make them commercially viable^3^. Increasing efficacy is most likely to come through the discovery of additional and relevant vaccine antigens.

The definitive, mammalian host of *F. hepatica* is orally infected by metacercariae on plants. Newly excysted juveniles (NEJ) emerge in the duodenum and migrate to the liver. Following a period of blood feeding and growth in the liver, they move to the bile ducts, where they obtain blood by puncturing the duct wall, undergo maturation, and produce eggs^4^. Although adult flukes are reproductively active and the major responsible for the pathology in mammalian hosts, NEJ are the cause of significant damage to host tissues when migrating from the gut lumen to the bile ducts^4^. During migration and development, parasites encounter different host tissues and macromolecules, dynamic physicochemical microenvironments, and host responses such as blood coagulation, complement activation, in addition to other innate and acquired immune responses^5^.

Parasite excretory/secretory (E/S) products are the collective material comprising proteins and other compounds secreted from the fluke’s gut, excretory pores and surface tegument; they are released by parasites within the host, or during *in vitro* culture^6^. These compounds play major roles in the parasite-host interface, since they are secreted during infection and protect the parasite from the host defensive responses^7^,^8^. Identifying E/S proteins secreted by parasites and understanding their associated functions within the host will improve our knowledge of their roles in parasite-host relationship, generating new insights into parasite biology.

The purpose of the present study was to perform a proteomic analysis of the intra-mammal stages of *F. hepatica*, comparing protein expression in E/S products from adults and NEJ, and somatic soluble proteins from NEJ. The results obtained here present new information for future studies on the discovery of diagnostic, therapeutic, and/or vaccine targets for this important foodborne disease.

## Results and Discussion

### Overview of the *F. hepatica* proteome

**Figure 1:**
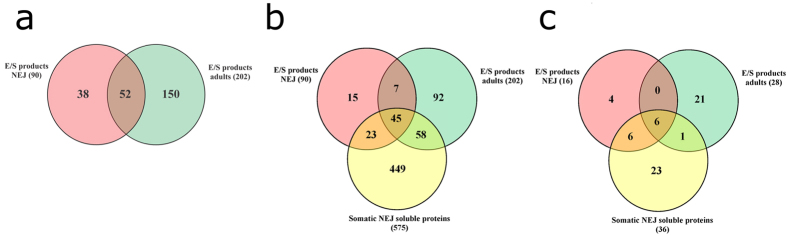
Distribution of proteins among *Fasciola hepatica* stages. (**a**) Comparison of *F. hepatica*-derived proteins identified in NEJ E/S products and adult E/S products. (**b**) Comparison of *F. hepatica*-derived proteins identified in NEJ E/S products, adult E/S products, and somatic NEJ soluble proteins. (**c**) Comparison of *Bos taurus*-derived proteins identified in NEJ E/S products, adult E/S products, and somatic NEJ soluble proteins. The overlap region between the circles shows proteins present in two or more stages.

**Figure 2:**
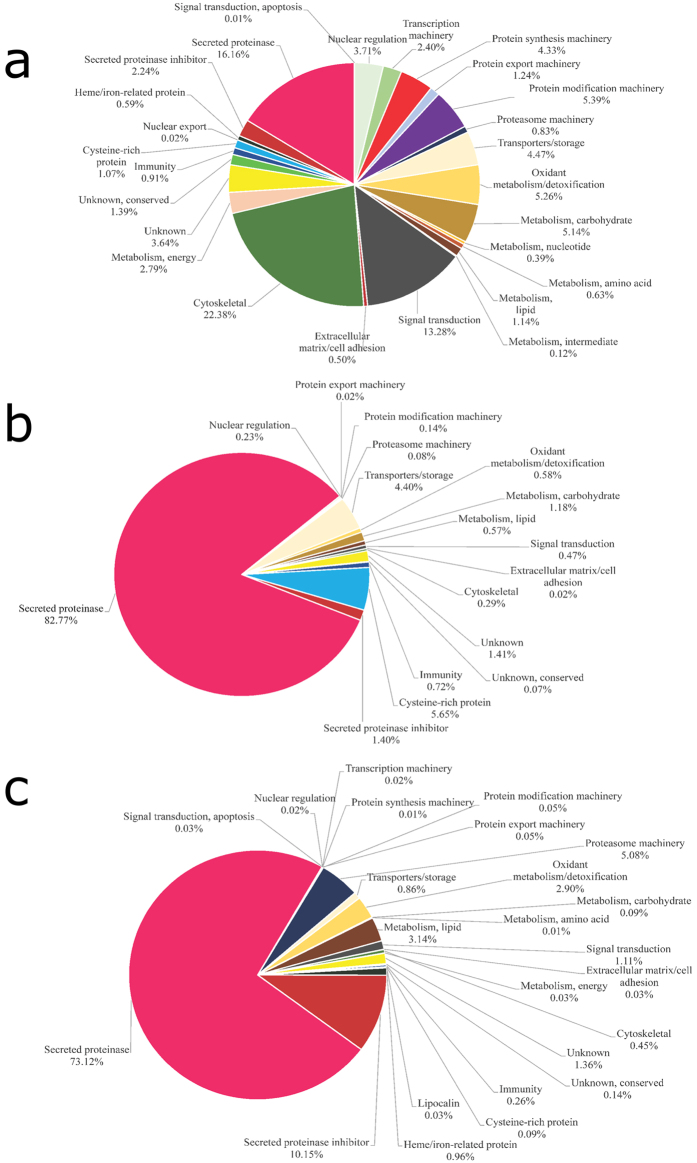
Functional classification of *F. hepatica*-derived proteins. Functional classification of *F. hepatica*-derived proteins identified in (**a**) somatic NEJ soluble proteins, (**b**) NEJ E/S products, and (**c**) adult E/S products. Pie charts represent the percentage of proteins found in each group with respect to normalized spectral counting (NSAF) for each sample.

In this study, a total of 689 *F. hepatica* proteins were identified (Fig. 1). This is the largest number of proteins identified so far for the intra-mammal stages of *F. hepatica*^7^,^9^,^10^,^11^. One concern about the E/S products produced *in vitro* is if they are actually secreted into the host tissue by flukes. Comparing NEJ somatic proteins with NEJ E/S products we could clearly observe that protein profiles are quite different (Fig. 2a,b); for instance some proteins, such as the cytoskeletal ones, are enriched in the NEJ somatic soluble fraction over the NEJ E/S products. Thus, demonstrating NEJ E/S products are indeed excreted/secreted by the parasite, and not the result of a rupture of the parasites during cultivation.

**Figure 3:**
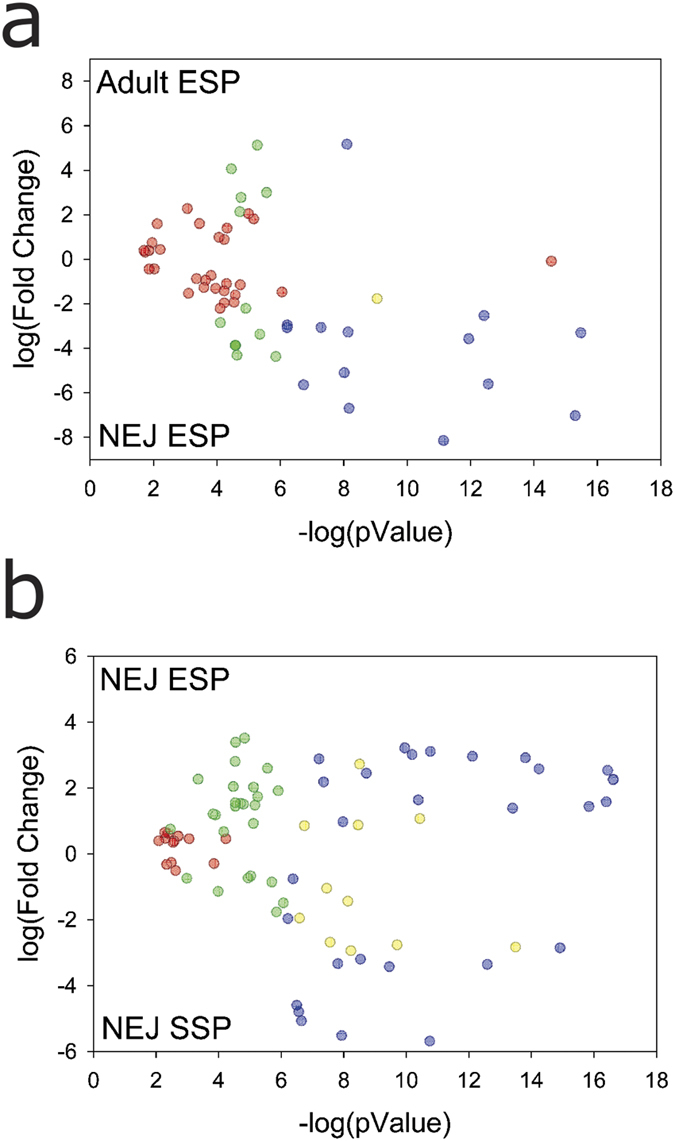
Volcano plot showing differentially expressed proteins. The volcano plot shows the results of differentially expressed proteins based on fold change versus t-test probability. (**a**) Plot obtained with the proteomic approach when comparing *F. hepatica*-derived proteins identified in E/S products from NEJ stage (NEJ ESP) with E/S products from the adult stage (Adult ESP) and (**b**) *F. hepatica*-derived proteins identified in E/S products from NEJ stage (NEJ ESP) with the somatic NEJ soluble proteins (NEJ SSP). Each protein is represented as a dot and is mapped according to its fold change on the ordinate axis (y) and t-test p-value on the abscissa axis (x). Proteins are represented by: (blue dot) if had an identification that satisfied both fold and statistical criteria; (yellow dot) had an identifications that was filtered out by the L-stringency; (green dot) had an identification satisfied the fold criteria but, most likely, this happened by chance; and (red dot) had identification did not meet the fold and p-value criteria.

In NEJ and adult E/S products, 240 proteins were identified: 90 from NEJ, 202 from adult flukes, and 52 proteins present in both stages (Fig. 1a). Of those shared proteins, a total of 14 (blue dots in Fig. 3a) were found to be differentially expressed in the two stages: the levels of 13 proteins were found to be higher in NEJ E/S products, whereas only one protein (a stefin) was found at higher levels in adult E/S products (Fig. 3a and Supplementary Table S1).

In the proteomic analysis of somatic soluble NEJ proteins, 575 proteins were identified, of which 68 proteins are shared with NEJ E/S products and 103 proteins are shared with adult E/S products (Fig. 1b). A core of 45 proteins is observed among the three samples (Fig. 1b). In terms of differential expression, we found that 29 proteins are differentially expressed comparing NEJ E/S products and somatic soluble NEJ proteins (blue dots at Fig. 3b). The expression levels of 17 proteins were found to be higher in NEJ E/S products, and 12 proteins were at higher concentration in somatic soluble NEJ extracts (Fig. 3b and Supplementary Table S2).

Host-derived proteins were identified in all samples. NEJ and adult E/S products presented 16 and 28 host-derived proteins, and 36 host-derived proteins were identified in somatic soluble NEJ extracts (Fig. 1c). Two host-derived proteins were found differentially expressed between NEJ E/S products and somatic soluble NEJ proteins (Fig. 3b and Supplementary Table S6).

### Proteinases

Relative expression analysis showed that the most abundant class of proteins in E/S products samples from NEJ and adults are proteinases, representing 83% and 73% of the total proteins in each sample, respectively (Fig. 2b,c).

**Figure 4:**
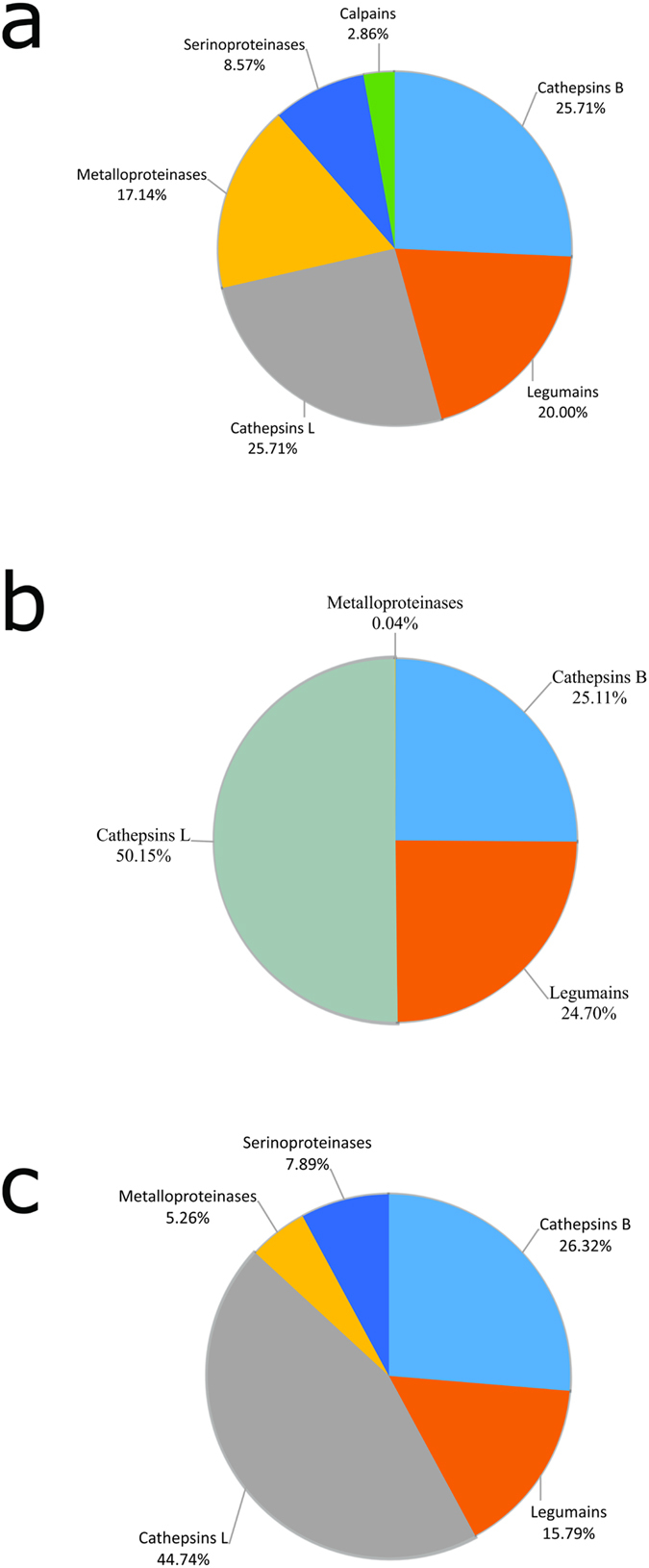
Functional classification of *F. hepatica*-derived proteinases. Proteinases identified in (**a**) somatic NEJ soluble proteins, (**b**) NEJ E/S products, and (**c**) adult E/S products were classified based on amino acid sequence similarity according to MEROPS definition. Pie charts represent the percentage of proteins found in each group with respect to normalized spectral counting (NSAF) for each sample.

In the somatic soluble NEJ sample, proteinases represented 16% of the total protein content (Fig. 2a). Cathepsins L and B represented 51% of the total proteinase content (25.7% each), followed by legumains (20%), metalloproteinases (17%), serine-proteinases (8%), and calpains (2.8%) (Fig. 4a). Seven metalloproteinases and three serine-proteinases were detected in the somatic soluble NEJ sample (Supplementary Table S3). Concerning leucine aminopeptidases, the analysis showed three matches in our database; however, contigs BN1106_s617B000566 and BN1106_s617B000567 represented, respectively, the C-terminal and N-terminal fragments of the same protein, *Fh*LAP. The third leucine aminopeptidase sequence (*Fh*LAP-2) is a novel protein among somatic soluble proteins from NEJ, and shows similarity with LAP-1 from other helminths.

In the NEJ E/S products sample, contrary to what was previously described^12^, the cathepsin L family was the most significantly represented group within cysteine proteinases, comprising 50% of all proteinases, followed by cathepsin B (25%) and legumain (24%) (Fig. 4b). We identified 12 cathepsin L proteins and 9 cathepsin B proteins (Supplementary Table S3, Supplementary Fig. S1 and Supplementary Fig S2). In addition, six legumains and one leucine amino peptidase (*Fh*LAP already described for *F. hepatica*)^13^ were found.

In the adult E/S products, the most abundant class of proteinases was also related to the cysteine proteinase family, with cathepsins L accounting for 44% of the total proteinase content, followed by cathepsins B (26%) and legumains (15%) (Fig. 4c and Supplementary Table S3). This is in agreement with previous reports^7^,^14^,^15^, however, in this study a higher number of cathepsin L-like proteins were identified. We found nine cathepsin L-, five cathepsin L1-, one cathepsin L2-, one cathepsin L3-, and one cathepsin L4-like proteinases (Supplementary Table S3). Furthermore, considering the 17 cathepsins L detected, it was identified at least one representative protein of each cathepsin L clade (CL1–CL4). Interestingly, clades CL3 and CL4 were previously described as NEJ-specific^16^. In adult E/S products, 10 cathepsins B, six legumains, three carboxypeptidases, one leucine aminopeptidase, and one dipeptidyl peptidase of the M24 family were identified (Supplementary Table S3).

All proteins that were found to be differentially expressed between NEJ and adult E/S products are proteinases, except for an alpha-glucosidase and CD59 (Fig. 3a and Supplementary Table S1). Eleven proteinases (legumains and cathepsins) exhibited higher levels in NEJ E/S products, in the range of 5- to 280-fold increase (Supplementary Table S1).

Cathepsins are secreted in the gut lumen following ingestion of host blood and liver tissue, to perform the digestion of host tissues and degradation of extracellular matrix, suggesting a role during invasion^17^. A role during the feeding process could be inferred based on the ability of cathepsin to degrade fibrinogen. Furthermore, since cathepsin L is able to cleave immunoglobulins, a possible function in the host immune system evasion could be assumed^18^,^19^. Liver flukes possess a blind-ending intestine, and the gut content is emptied by regurgitation^7^. Accordingly, proteinases once released may carry out additional important functions for the parasite-host relationship, since liver fluke cathepsins L can cleave interstitial matrix proteins such as fibronectin, laminin, and native collagen^17^. More recently, it was proposed that the secreted cathepsin L may be involved in suppression and/or modulation of Th1 immune responses and induction of non-protective host Th2 responses^20^.

### Proteinase inhibitors

The proteinase inhibitors identified among NEJ E/S products represented 1% (four different proteins) of the total protein content (Fig. 2b), two of which were Kunitz-type inhibitors, one was a stefin, and one an inhibitor belonging to the I63 family (Supplementary Table S3). The proteinase inhibitors in the adult E/S products sample represented about 10% of the total protein content, being the second most-represented class (Fig. 2c). Three cysteine proteinase inhibitors and seven serine proteinase inhibitors (five serpins and two Kunitz-type inhibitors) were identified. In the NEJ somatic soluble sample, 2% of total proteins were proteinase inhibitors (two cystatins, one stefin, five serpins and one Kunitz-type inhibitor) (Fig. 2a).

Since proteinases are important to accomplish physiological processes and have a wide spectrum of activity within the parasites, they have to be tightly regulated, or else they could be harmful to both parasite and host^21^. In addition, it has been demonstrated that proteinase inhibitors may modulate host defenses against ecto- and endoparasites^22^,^23^.

Kunitz-type inhibitors are low-molecular-weight, competitive serine proteinase inhibitors that behave in a substrate-like manner and form stable complexes with their target proteases^24^. There is to date only one Kunitz-type inhibitor described for *F. hepatica (Fh*-KTM)^25^. It was described in the adult stage only, and its activities include trypsin inhibition and suppression of pro-inflammatory cytokine production by dendritic cells^23^. In our study, another Fh-KTM-like protein was found also in the E/S products from NEJ and adults (Supplementary Table S3), sharing 44% identity in amino acid sequence with Fh-KTM (data not shown).

Five serpins (serine protease inhibitors) were identified in adult E/S products and somatic soluble NEJ (Supplementary Table S3). Serpins are irreversible inhibitors of serine proteinase mediators of host defense pathways^26^. Several serpin-encoding cDNAs from parasites have been cloned and characterized^27^,^28^, although no data has been published about these proteins from *F. hepatica*. Some studies have shown that parasite-encoded serpins are functional inhibitors likely associated with evasion of host defenses, displaying anticoagulant and immunomodulatory properties^29^,^30^, a characteristic that could be important for *F. hepatica* establishment and survival in the host.

Cystatin is a superfamily of cysteine protease inhibitors. In the adult E/S products and somatic soluble NEJ proteins, one stefin (family-I cystatin) and two cystatins (family-II cystatins) were identified. In NEJ E/S products only one stefin was identified (Supplementary Table S3). This stefin represents the only up-regulated protein in adult E/S products, 35.8-fold higher when compared with NEJ E/S products (Supplementary Table S1 and Supplementary Table S5). It shares 93% similarity with the one described in *F. gigantica* adult E/S products^31^. This stefin was partially found in complex with cathepsin L, suggesting a role in the regulation of cysteine proteinases activity and/or protection against extracellular proteolytic damage to the parasite intestinal tissue^31^.

### Hemoglobin metabolism and heme-related proteins

In adult E/S products and somatic soluble NEJ extract, five proteins were identified which are related to hemoglobin metabolism, including: one myoglobin, three ferritins and MF6p/FhHDM-1^7^,^9^,^32^ (Fig. 2a,c and Supplementary Table S3). Two of the ferritins are novel protein descriptions for *F. hepatica*. The main source of nutrients necessary for the survival of hematophagous parasites in the host comes from the digestion of host hemoglobin, by proteolytic proteinases within the lumen of the parasite gut^33^. Hemoglobin degradation releases the iron-containing prosthetic group heme, which is toxic when free in biological systems^34^, and has pro-inflammatory properties^35^. To avoid this, blood feeders have developed strategies for detoxifying excess heme, such as the crystallization of heme into hemozoin^36^. It is assumed that adult *F. hepatica* releases heme and other waste molecules directly in the biliary ducts^37^, but its capacity to form hemozoin remains unknown, although it has been described for other trematodes^38^. Therefore, heme-binding proteins such as MF6p/FhHDM-1 and myoglobin could be used during adult *F. hepatica* blood feeding process as free-heme ligands, as a way to release excess heme and prevent heme-induced inflammation. On the other hand, heme is also a cofactor required by most living organisms to form heme proteins, which are involved in several biochemical processes^39^. Most eukaryotic cells have enzymes that enable complete *de novo* heme biosynthesis, but it is generally accepted that hematophagous parasites have a total or partial loss of the *de novo* heme biosynthesis pathway^34^. These parasites have had to develop heme intake, transport, and storage mechanisms, while protecting their tissues from the putative toxic effects of such molecules^40^. Proteins that belong to this class were not identified in NEJ E/S products. Little is known about the diet of NEJ; it is suggested to comprise mainly host tissue cells, although some blood could be also ingested^41^. Therefore, the identification of heme-binding proteins exclusively in somatic soluble NEJ extract (but not in NEJ E/S products) indicates that they may contribute in the storage and supply of heme to NEJ metabolism, but are not secreted in NEJ E/S products as a way to release excess host heme.

Myoglobin (Mb) is a heme-protein described in trematodes which may have a role in host-parasite interactions, but its physiological functions are still in debate^42^. Globins of non-vertebrate species show higher variability in their structures, which might reflect their adaptations to specific functions, when compared with their vertebrate homologs^43^. Indeed, adult parasitic helminths live mainly in a semi-anaerobic environment, and their hemoglobin-like proteins display such a high oxygen affinity that they are unlikely to serve only as O_2_ transporters. In platyhelminthes, other functions for these proteins have been described, such as oxygen scavenging, heme reserve for egg production, and NO dioxygenase^44^,^45^. Thus, they are functional molecules with considerable affinity for oxygen, and their role in oxygen transport and supply should be critical under the low-oxygen conditions typical of the host microenvironment. Consumption of oxygen is associated with energy metabolism; in *F. hepatica*, the Krebs cycle, which is by far the major energy-yielding pathway of NEJ and migrated juvenile flukes, is gradually replaced by aerobic acetate formation, and finally by anaerobic dismutation reactions in adult liver flukes^46^.

### Energy Metabolism

A total of 16 proteins with a role in energy metabolism were found, representing 3% of the total protein content in somatic soluble NEJ extracts (Fig. 2a and Supplementary Table S3). Also, one protein of this functional class, a glyceraldehyde-3-phosphate dehydrogenase, was found in the adult E/S products sample. This could suggest that energy requirements differ between stages of the same parasite.

A cytochrome C proximal sequence identified in the soluble NEJ proteins sample presents high similarity with cytochrome C from other trematodes such as *Schistosoma japonicum* and *Opisthorchis viverrini* (78% and 76% of sequence similarity, respectively; data not shown). Cytochrome C oxidase is a large transmembrane protein complex and it is the last enzyme in the mitochondrial respiratory electron transport chain, a crucial process for hypoxic response in aerobic organisms. However, hypoxia adaptation mechanisms in mitochondria are still poorly understood^47^. Moreover, the high homology with a Tibetan bird cytochrome C protein suggests a similar role in *F. hepatica*. They are able to improve physiological performance by enhancing oxygen transport capacity, and have yielded important insights into the genetic basis of adaptation involving the critical oxygen carrier, hemoglobin^48^.

Among somatic soluble NEJ proteins we identified one acetate/succinate CoA transferase (Supplementary Table S3) previously characterized in the mitochondria of adult fluke^49^. As discussed above, the availability of oxygen is limited during all or part of the parasite life cycle; therefore, it requires ATP-synthesis pathways that are independent of O_2_ as the terminal electron acceptor. The formation of acetate as an end-product from acetyl-CoA is a metabolic route present in parasites that survive in hypoxic or anoxic habitats^50^. Formation and excretion of acetate as an end-product of energy metabolism are catalyzed by a cytosolic acetyl-CoA synthetase (ACS) or by an organellar acetate:succinyl CoA-transferase (ASCT)^51^. In *F. hepatica*, ASCT catalyzes these reactions, indicating that acetate is synthesized in NEJ through the ASCT pathway. Given that acetate is not formed by mammalian hosts, acetate production might harbor novel targets for the development of anti-parasitic drugs.

### Cysteine-rich proteins

In this group we found proteins belonging to the superfamily CAP (Cysteine-rich secretory protein, Antigen 5, and Pathogenesis-related-1 proteins), and to the CRP (Cysteine-rich protein) family. The CAP superfamily members are found in a remarkable range of organisms, and have been described in helminths^52^, as well as in other parasites^53^. These proteins are generally secreted with a broad range of functions, including: regulation of the extracellular matrix, proteases or protease inhibitors, ion channel regulation, reproduction, and cellular adhesion^54^.

In the NEJ E/S proteins sample, we identified four proteins (5% of the total protein content) corresponding to the peptidase inhibitor 16 subfamily (PI16). They share similarity with *Clonorchis sinensis* PI16 proteins, and two of them were also detected among somatic soluble NEJ proteins (1% of the total proteins) (Fig. 2 and Supplementary Table S3). However, PI16 subfamily is not well characterized, and it remains to be established whether these proteins have proteinase inhibitory activity.

Concerning the adult E/S products, two proteins of the glioma pathogenesis related-1 (GLIPR1) subfamily were identified, both of which are novel sequences with similarity to *C. sinensis* proteins. GLIPR1 proteins represent the second best-characterized CAP subfamily in mammals. In a previous transcriptomic analysis, some proteins of this subfamily were predicted *in silico* for different trematodes^55^. In that study, the authors predicted two proteins with double cysteine domains, up-regulated in juvenile stages of *F. hepatica*, and other nine proteins with only one domain. Nonetheless, our work is the first to experimentally identify GLIPR1 proteins in *F. hepatica* secretome. In *Ancylostoma caninum*, proteins belonging to this class are related to neutrophil and platelets aggregation inhibition, and participate in gastrointestinal hemorrhage and iron deficiency anemia^56^.

In the somatic soluble NEJ proteins, one protein was identified belonging to the CRP family. These proteins mediate protein–protein interactions and are important for cell differentiation, cytoskeletal remodeling, and transcriptional regulation^57^. Further analysis and experimental data should help to understand the biological function of all these proteins in parasites.

### Transport/Storage

Transport proteins represented 4% of the total protein content in both samples from NEJ (Fig. 2a,b). On the other hand, they represented less than 1% of the total proteins in adult E/S products (Fig. 2c). In NEJ E/S products, the majority of the transport proteins found belong to the cubilin family (Supplementary Table S3). Cubilins are peripheral membrane glycoproteins without transmembrane segments, having CUB domains that indicate potential binding sites for various ligands^58^. These proteins, in complexes with megalin or RLP2, could work as endocytic receptors for a variety of proteins, including hemoglobin, albumin, ferritin, vitamin-carriers, and lipoproteins^59^. In a recent study in mice, cubilin was apparently responsible for binding ferritin, while megalin was the protein responsible for iron uptake by the cells^60^. Megalin was not found in our samples; the lack of this protein in *F. hepatica* possibly implicates cubilin binding as a way to excrete heme from hemoglobin digestion without generating an immune response from the host. In the adult E/S sample, we found some proteins related to exosomes^61^, ABC transporters, and calmodulins, as well as one glucose transporter.

### Lipid Metabolism

Lipid metabolism is the least-studied metabolic pathway in *F. hepatica*, as the total lipid content of adult fluke represents only the 1.2% of its dry tissues^62^. From this functional class, 13 proteins were identified in adult E/S products, 3 in NEJ E/S products and 11 in the soluble components of NEJ (Supplementary Table S3). In adult E/S products, the most abundant components were the fatty acid binding protein type 2 (*Fh*FABP), a saposin-like protein, and a Niemann-Pick protein. The FABP detected has a 191-aa-long sequence that matches *F. hepatica* FABP2; however, there was no match in our data for FABP1 and FABP3, in contrast to other proteomic studies^7^,^9^. Two saposin-like molecules, *Fh*SAP-1 and *Fh*SAP-2, were already described for *F. hepatica*^63^,^64^ and a third saposin, named *Fh*SAP-3, was described for *F. gigantica*^65^. In accordance with those previous reports, three saposin-like proteins were identified in the E/S products of adult flukes, but only one of them, *Fh*SAP-1, was observed in NEJ samples. Other proteins found were Niemann-Pick proteins, which are involved in the transport of cholesterol within the late endosomal-lysosomal compartment^66^. Besides the first description of the genes in *Caenorhabditis elegans*, the NPC2 protein has been found as one of the most abundant proteins in *Schistosoma mansoni* eggs^67^, and also present in extracellular vesicles of adult *F. hepatica*^61^. Our results are in accordance with the previous reports. However, from the five Niemann-Pick proteins detected in adult E/S products, one was also found at high abundance in NEJ E/S products.

In the somatic soluble NEJ proteins we also identified an enzyme related to the arachidonic acid pathway: the prostamide/prostaglandin F synthase, which includes a thiolase domain in its sequence (Supplementary Table S3). To date, this proteins have been described only in protozoan parasites such as *Trypanosoma brucei*, which produces prostaglandin F2α^68^. Prostaglandins of the 2-series are synthesized from arachidonic acid, and their function in mammals is to modulate different physiological processes^69^. The role of this prostamide/prostaglandin F synthase in *F. hepatica* is unknown, but since they produce PG_2_, we could hypothesize a role in parasite-host relationship, as immunomodulators, in vacuole formation, or as signal transductors for apoptosis^70^,^71^.

### Miscellaneous

The alpha-glucosidase found in our study is up-regulated in NEJ E/S products, when compared to adult E/S products (Fig. 3 and Supplementary Table S5). Alpha-glucosidase is a protein involved in the hydrolysis of starch and disaccharides to glucose, which has been reported in other blood-sucking species in association with hemozoin formation, but so far never described in helminth species^72^. Glucosidase activity was described in adult E/S products from *F. hepatica*^73^; however, β-glucosidase was not found in our analysis. As long as it is assumed that *F. hepatica* does not have the capacity to form hemozoin, we suggest that the role of alpha-glucosidase in this parasite could be as part of a strategy to overcome heme toxicity in the host. Furthermore, alpha-glucosidase could take part in the conversion and transport of complex sugars into simpler ones, and their presence in the E/S products could mean that gastrodermis also has a role in glucose uptake.

We were also able to find proteins related to the proteasome machinery in the samples (Fig. 2). In the adult E/S products, we identified two proteins that represented 5% of the total protein (Fig. 2c), and were among the most abundant proteins in the sample (Supplementary Table S3). Concerning their presence in NEJ samples, we identified 2 proteins in NEJ E/S products and 15 proteins in somatic soluble NEJ proteins (Fig. 2 and Supplementary Table S3). The proteasome and its associated proteins have not been well studied in trematodes, but in *S. mansoni* it was demonstrated the presence of a functional proteasome^74^. Protein ubiquitination is responsible for the regulation of various biological processes through covalent modification of proteins and transcription factors. During its life cycle, the parasite is exposed to physical and chemical stress. Free-living stages are subjected to threats, such as chemical pollutants, variations in temperature, and solar radiation, while parasitic stages are exposed to oxidative stress and the host immune system. The proteasome represents the main cytoplasmic proteolytic machinery for the degradation of damaged proteins^75^, and is responsible for the maintenance of protein homeostasis during oxidative stress^76^. Apart from these various antioxidant defense systems, we also identified superoxide dismutase, peroxiredoxins, glutathione peroxidase, cytochrome C, glutathione S-transferase, glutathione reductase, and thioredoxin glutathione reductase in *F. hepatica* (Supplementary Table S3). These findings suggest that the proteasome machinery is important across different intra-mammal stages, though it seems to have a fundamental role in the adult fluke.

Only one lipocalin protein was identified in adult E/S products (Supplementary Table S3). Lipocalins are proposed to be members of a superfamily of proteins named calycins, which comprises proteins with a β-barrel that binds small, lipophilic ligands^77^. This superfamily includes fatty acid binding proteins (FABP), avidin, metalloproteinase inhibitors, and triabin^78^. There is still no evidence of the function of lipocalins in trematodes, but they are a well-studied family of proteins in other blood-sucking arthropods, like ticks^79^. In ticks, lipocalins are present in saliva, and part of their role could be that of antihemostatic and immunomodulatory molecules, helping to maintain parasite attachment to host tissues and to overcome the host immune system. Like ticks, flukes are blood feeders which depend upon attachment to host tissues, thus lipocalins could perform a similar function in *F. hepatica.*

Other novel proteins found in the somatic soluble NEJ sample are the actin-associated proteins (Supplementary Table S3). It is known that several actin-associated proteins are required to maintain the high rates of actin filament disassembly and turnover that drive biological processes. Rapid cycles of actin assembly and disassembly require actin-binding proteins, including beta-thymosins, actin-binding competitor profilin^80^, and the depolymerization factors cofilin^81^ and coronin^82^. Their role may be associated with vesicular trafficking in the tegumental syncytium of NEJ, as well as to other intracellular events in muscle tissue or nervous system development which require formation and/or depolimerization of actin. In particular, coronins are highly conserved proteins involved in actin dynamics across eukaryotic systems, including filament binding and bundling. They generally bind to F-actin and apparently are involved in proliferation, locomotion, and phagocytosis^83^. A major actin binding protein, called beta-thymosin, was described in *Trichinella spiralis* and it is so far the best-studied actin-associated protein from helminth parasites^84^. It sequesters beta-actin to regulate its polymerization, functions as an angiogenic factor which is up-regulated during *T. spiralis* nurse cell formation, and is co-localized with beta-actin within infected host muscle^84^. Nevertheless, the role of those proteins in *F. hepatica* biology remains to be elucidated. However, it comes as no surprise that NEJ possesses this battery of actin-binding proteins that might be used in morphogenesis, tissue remodeling, and/or in exocytosis/endocytosis events during its development into the larger immature stages.

Furthermore, proteins with potential roles in exosomal membrane structure were identified in both, adult and NEJ E/S products samples (Supplementary Table S1)^10^,^85^. This is the first identification of exosome-related proteins among NEJ E/S products. Considering all the new data about the components of E/S products, including host and exosomal proteins, maybe it is time to formally revise the definition and composition of helminth E/S products.

### Host-derived proteins

**Figure 5:**
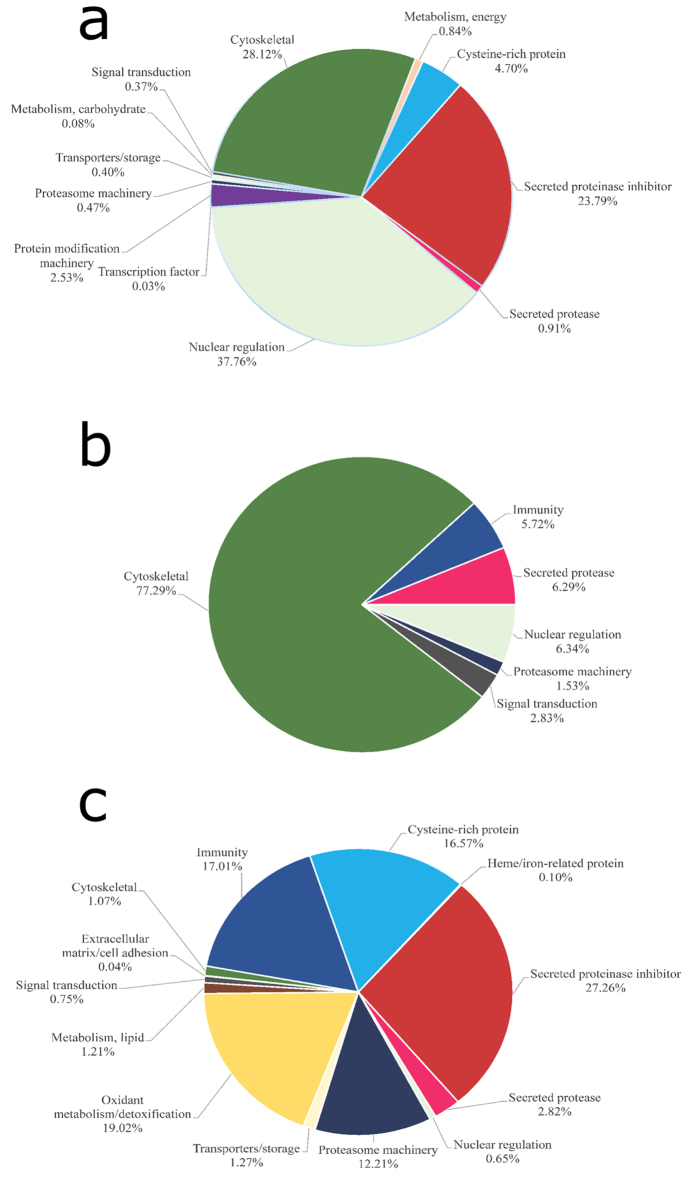
Functional classification of *Bos taurus*-derived proteins. Functional classification of *B. taurus*-derived proteins identified in (**a**) somatic NEJ soluble proteins, (**b**) NEJ E/S products, and (**c**) adult E/S products. Pie charts represent the percentage of proteins found in each group with respect to normalized spectral counting (NSAF) for each sample.

Host-derived proteins (based on a *B. taurus* database) were identified in all samples, including 16 proteins in NEJ E/S products, 28 proteins in adult E/S products, and 36 proteins in somatic soluble NEJ extract (Fig. 1c and 5 and Supplementary Table S4). Based on relative abundance, most of them are blood-related proteins. During adult E/S products collection, only adult flukes that had visibly emptied their guts were used; however, they may still have had mammal blood proteins in them. Interestingly, we were not able to find albumin or hemoglobin, the major proteins in blood, proposing that the host-derived proteins identified here are more than contamination agents due to parasite regurgitation. In agreement with this hypothesis, some of the host proteins described here have been found in secreted exosome vesicles of *F. hepatica*^10^, suggesting that their presence in parasite secretions may be a real and common recycling system, not a result of contamination during sample collection. This finding is in accordance with previous studies that investigated parasite secretion from other blood-feeding parasites^79^. It remains to be clarified whether these proteins are returned to the host as intact proteins or products of partial hydrolysis.

Host-derived proteins with a role in regulation of host defense pathways against parasites were detected in adult E/S products, including: antithrombin III (thrombin inhibitor), alpha-1-antitrypsin and serpin B1 (neutrophil elastase inhibitor), immunoglobulin (humoral response), alpha-2-macroglobulin (blood coagulation), fibrinogen (blood coagulation and platelet aggregation), and kininogen-2 (inflammation and coagulation) (Supplementary Table S4). Liver fluke adults establish contact with the host immune and hemostatic systems, and have to evade them in order survive. Thus, one possible explanation for the presence of host-derived proteins in parasite secretion is that parasites recycle pivotal host proteins in order to subvert their role in the host and/or use host proteins in specific parasite physiologic systems. Alpha-1-antiprotease, serpin B1, and antithrombin III were identified in adult E/S secretions. These proteins regulate proteinases such as neutrophil elastase and thrombin, and thus it would be interesting to find out whether these host proteins have the potential to inhibit their own serine proteinases. In turn, the presence of immunoglobulin chains could be explained as a parasite self-defense system, in a process leading to removal of host immunoglobulins. In addition, a recent study demonstrated the use of host-derived transferrin in the tick *H. longicornis*. This host-derived transferrin moves particularly from the midgut via hemolymph to the tick’s ovary, raising the possibility that it functions as an iron source in this organ^86^

Host-derived proteins were also found in NEJ samples (Fig. 5a,b and Supplementary Table S4). This is a quite interesting finding, since NEJ hatched from metacercariae *in vitro* are not expected to have host proteins as contaminants. In the NEJ stage, the host-derived proteins identified are mainly represented by cytoskeletal and nuclear proteins such as histone H2A, histone H4, actin-2, tubulin, and HSP 70. These proteins are largely conserved across species, and these signals could have been derived from parasite proteins instead. Accordingly, to further control the origin of the putative host-derived proteins identified, we compared these host protein sequences with proteins from *F. hepatica* (data not shown). Indeed, a great part of the detected host proteins is highly conserved. For example, mammalian histone is 89% identical to *F. hepatica* histone, while host actin and tubulin are 93% and 92% identical to the liver fluke proteins, respectively. Many proteins show similarity >65% to liver fluke proteins, and thus their origin cannot be determined. The mammalian keratin found in all samples could represent a major contamination from laboratory manipulation^87^.

Despite reports of host-derived proteins in parasite exosomes^85^, this remains a neglected issue in the study of parasite biology. The demonstration of these proteins in the present work raises several questions to be further explored, and may reveal novel insights into parasite-host relationship.

## Conclusions

The purpose of this study was to characterize the protein composition of mammal-related stages of the endo-parasite *F. hepatica*. The liver fluke secretes/excretes proteins which are qualitatively and quantitatively different during its intra-mammal life stages, likely reflecting different locations within the host. The use of LC-MS/MS approach allowed us to identify proteins previously found in other proteomic studies, as well as other proteins described here for the first time in *F. hepatica*.

Comparing our data with previous studies, the proteomic strategy used here allowed us to identify proteins that are not abundant in the samples (Fig. 2, Supplementary Tables S3 and S4). We identified 575 proteins in the soluble NEJ extract, 90 proteins in the E/S products from NEJ, and 202 proteins in adult E/S products. In a recent study combining transcriptomic and proteomic data, 160 secreted proteins were predicted in the transcriptomic approach: however, only 22 proteins were found in adult flukes and 29 proteins were detected in NEJ secretions using a 2-DE proteomic approach^7^. In another study on secreted proteins from adult fluke, 29 out of 60 prominent proteins were identified, also using a 2-DE approach^14^, while in an study using LC-MS/MS, only 16 out of 54 proteins from NEJ were suggested to be secreted^88^. In a study from 2010 on proteins expressed by freshly excysted parasites, or NEJ, the authors found a total of 40 *F. hepatica* proteins using LC–MS/MS^11^. In 2011, a study on the adult fluke secretome identified 63 proteins, whereas in a recently published article the authors identified 69 proteins in *F. hepatica* extracellular vesicles^9^,^10^. Comparing these studies, we found that all share a common group of proteins, but with some differences (Supplementary Table S7). These differences could maybe be explained based on the different technical procedures and materials used in each study. Another point to keep in mind is that, even though the *F. hepatica* adults used in these studies belong to the same species, they are subject to different environments (weather conditions, soil, cattle, etc.), which may influence the amount and variety of secreted proteins.

In general, another concern is about the biological relevance of the proteins identified by a proteomic analysis. In a previous proteomic study of bile from *F. hepatica*-infected animals, had shown that the major *F. hepatica* proteins present in the host were proteases, as well as, in the *in vitro* culture^15^. In addition, it was demonstrated that E/S products can be used for diagnosis of human fascioliasis^89^,^90^. Therefore, proteins identified in the present study are physiologically relevant since them, besides being identified in other proteomic studies, were also previously validated as secreted proteins in natural infections.

## Methods

### Ethical statement

This study was conducted in accordance to the ethic and methodological aspects preconized by the International and National Directives and Norms by the Animal Experimentation Ethics Committee of the UFRGS. The protocols were approved by the Comissão de Ética no Uso de Animais - CEUA and UFRGS (No. 28309). Cattle livers were collected from a local abattoir immediately after slaughter. Natural liver fluke infections were diagnosed at abattoir by independent trained meat inspectors and the biological material discarded by the local abattoir protocol. The abattoir authorized by the Ministry of Agriculture and Fisheries of Uruguay (MGAP), complies with the National Animal Welfare Act No. 18471 of 2009 law of protection, welfare and possession of animals, regulated by: Decree No. 62/014 14.03.2014; and with the good animal welfare practices concerning transport and slaughter of cattle and sheep, prepared by the Technical Group of the Directorate General of Livestock Services (DGSG-MGAP) in 2005, according with the recommendations of the 73rd General Session of the OIE-World Organisation for Animal Health on 27 May 2005.

### Preparation of *F. hepatica* E/S products

*F. hepatica* metacercariae (Oregon strain) were purchased from Baldwin Aquatics Inc. (Monmouth, OR, USA). In order to obtain E/S products, metacercariae were activated *in vitro* to allow eclosion of NEJ, as previously described^91^, and the process was monitored under a binocular stereo zoom microscope. The emerging active parasites were washed with sterile PBS, then 600 to 800 NEJ were picked up and incubated at 37 °C, 5% CO_2_ in 1 ml of sterile culture medium, composed of RPMI 1640 supplemented with 30 mM HEPES pH 7.2, 2% glucose and 10% antibiotic/antifungal mixture (Penicillin/Streptomycin/Amphotericin B Mix). NEJ were maintained alive in culture for 48 h; every 12 h, the supernatant containing the E/S products was collected under a security cabinet with laminar flow, and replaced with fresh sterile medium. During medium exchange, NEJ integrity was evaluated by motility visualization. All samples were pooled under laminar flow, syringe-filtered through sterile filter (0.22 μm), processed for buffer exchange against sterile PBS, and concentrated using centrifugal filter units of 3,000-Da cut-off. Aliquots were lyophilized and stored at −80 °C until use.

Adults flukes (n = 50) were collected from the bile ducts of four different infected cattle from a local abattoir in Montevideo, Uruguay, and E/S products were obtained cultivating flukes into 1 mL of culture medium per fluke following incubation at 37 °C. After 3 hours, a total of 50 mL of supernatant was collected and filtered esterilezed^19^. The product was stored at −80 °C until use. To verify the absence of contaminating proteins in the samples, the taurocholic acid used during the excystment procedure was analyzed by LC-MS/MS as a control.

### Preparation of *F. hepatica* NEJ soluble extracts

NEJ (n = 400) were collected (as described under item 2.1) in a sterile 1.5-mL tube, washed 3 times in PBS with protease inhibitor cocktail, and sonicated for 5 min with 60-s bursts at 20% power followed by 30-s pauses, using a tissue homogenizer. The homogenate was centrifuged at 48,000 g for 10 min at 4 °C. The obtained supernatant was stored at −80 °C until use. Protein extracts were quantified at 280 nm in a Nanodrop 1000 spectrophotometer (Thermo Fisher Scientific, USA).

### Protein digestion and sample preparation

Protein samples were digested in solution with trypsin. Samples were diluted in 8 M urea/0.1 M Tris, pH 8.5, reduced with 5 mM Tris (2-carboxyethyl) phosphine hydrochloride, and alkylated with 25 mM iodoaceamide. Proteins were digested overnight at 37 °C in 2 M urea/0.1 M Tris pH 8.5, 1 mM CaCl_2_ with trypsin at a final ratio of 1:20 (w/w) (enzyme:substrate). Digestion reactions, at a final protein concentration of 0.15 μg/mL, were quenched with formic acid (5% final concentration) and centrifuged at 17,000 g for 5 min at 4 °C for removal of debris.

### Pre-columns and analytical columns

Reversed phase pre-columns were prepared in 250 μm ID/360 μm OD capillaries with a Kasil frit at one end. Pre-columns were packed in-house with 2 cm of a 5-μm ODS-AQ C18 particle slurry in methanol. Analytical reversed phase columns were prepared by pulling a silica capillary (100 μm ID/360 μm OD) to a 5-μm ID tip, and packing 20 cm of the above mentioned particles directly onto the pulled tip. Reversed phase pre-columns and analytical columns were connected using a zero-dead volume union.

### LC-MS/MS

Peptide mixtures were analyzed by nanoflow LC-MS using an Easy NanoLC II coupled to a Q Exactive mass spectrometer (Thermo Fisher Scientific, USA). Solutions A and B consisted of 5% acetonitrile/0.1% formic acid and 80% acetonitrile/0.1% formic acid, respectively. The flow rate was set to 400 nL/min. Protein samples (1.5 μg per injection) were separated in 155-min chromatographic runs, as follows: 1–10% B in 10 min, 10–40% B in 100 min, 40–50% B in 10 min, and 50–90% B in 10 min. The column was held at 90% B for 10 min, then brought to 1% B and re-equilibrated prior to the next injection. Peptides eluted from the analytical column were electrosprayed directly into the mass spectrometer.

The mass spectrometer was operated in a data-dependent mode, collecting a full MS scan from 400 to 1,200 m/z at 70,000 resolution and AGC target of 1 × 0^6^. The 10 most abundant ions in each scan were selected for MS/MS at 17,500 resolution, with AGC target of 2 × 10^5^, and an underfill ratio of 0.1%. Maximum fill times were 20 ms and 120 ms for MS and MS/MS scans, respectively, with dynamic exclusion of 15 s. Normalized collision energy was set to 25.

### Data Analysis

Tandem mass spectra were extracted from Thermo RAW files using RawExtract 1.9.9.2 92, and searched with ProLuCID^93^ against a non-redundant database containing coding sequences from *Fasciola hepatica* genome^94^ (33,454 entries), concatenated with a *Bos taurus* Uniprot reference database (23,804 entries), in addition to reverse sequences of all entries. Searches were done using Integrated Proteomics Pipeline (IP2, http://www.integratedproteomics.com). The search space included all fully-tryptic and half-tryptic peptide candidates. Carbamidomethylation of cysteine was used as static modification. Data were searched with 50-ppm precursor ion tolerance and 20-ppm fragment ion tolerance.

The validity of the peptide spectrum matches (PSMs) generated by ProLuCID was assessed using Search Engine Processor (SEPro) module from PatternLab for Proteomics platform^95^. ProLuCID XCorr, DeltaCN, DeltaMass, Z-score, number of matched peaks, and secondary rank values were used to generate a Bayesian discriminating function. A cut-off score was established to accept a false discovery rate (FDR) of 1% based on the number of decoys. A minimum sequence length of six residues per peptide was required. Results were post-processed to only accept PSMs with precursor mass error <10 ppm.

A Volcano plot was generated by pairwise comparison between NEJ and adults E/S products, and between NEJ E/S products and NEJ somatic soluble extract, using the PaternLab’s TFold module^95^. NSAF (normalized spectral abundance factor) was used for data normalization^96^. NSAF for a given protein is the number of spectral counts (SpC) identified for that protein, divided by the protein’s length (L), divided by the sum of SpC/L of all protein in the experiment. The following parameters were used to select differentially expressed proteins: proteins were grouped by maximum parsimony, spectral count data were normalized using NSAF values, and two nonzero replicate values were required for each condition (at least two out of three replicates). A BH q-value was set at 0.02 (2% FDR). A variable fold-change cut-off for each individual protein was calculated according to the t-test p-value using an F-stringency value automatically optimized by the TFold software. Low-abundance proteins were removed using an L-stringency value of 0.4.

### Protein functional annotation and classification

To manually curate *F. hepatica* and *B. taurus* protein database annotation, BLASTP searches against several databases were performed. To functionally classify the protein sequences, a program developed and provided by Dr. José M. C. Ribeiro was used^97^. The functionally annotated catalog for each dataset was manually curated and plotted on a hyperlinked Excel spreadsheet (Supplementary Tables S3 and S4).

### Relative abundance and graphical visualization

Proteomic profiles were compared across samples as functional classes or individual proteins. To determine the relative abundance of proteins, NSAF was used in a label-free relative quantification approach^98^. Mean NSAF values from the two or three replicates were determined and combined according to functional class, and then divided by the total NSAF for the respective sample. NSAF as an index for relative protein abundance was input in Microsoft Excel as percentage of the total NSAF for respective samples, and visualized on pie charts according to protein classes.

### Data availability:

The mass spectrometry proteomics data have been deposited to the ProteomeXchange Consortium via the PRIDE partner repository with the dataset identifier PXD003214.

## Additional Information

**How to cite this article**: Di Maggio, L. S. *et al*. Across intra-mammalian stages of the liver fluke *Fasciola hepatica*: a proteomic study. *Sci. Rep.* **6**, 32796; doi: 10.1038/srep32796 (2016).

## Supplementary Material

Supplementary Information

Supplementary Table S3

Supplementary Table S4
