# Supplementary material for: Across intra-mammalian stages of the liver f luke Fasciola hepatica: a proteomic study
Source: Sci Rep. 2016 Sep 7;6:32796. doi: 10.1038/srep32796 (PMC5013449; doi:10.1038/srep32796)
Supplement: Supplementary Information [file srep32796-s1.pdf]

Across intra-mammalian stages of the liver fluke Fasciola hepatica: a proteomic study

Sánchez Di Maggio, L.; Tirloni, L.; Pinto, A.F.M.; Driedrich, J.K.; Yates III, J.R.; Benavides, U.; Carmona, C.; da Silva Vaz Jr; Berasain, P.

Supplementary Information.

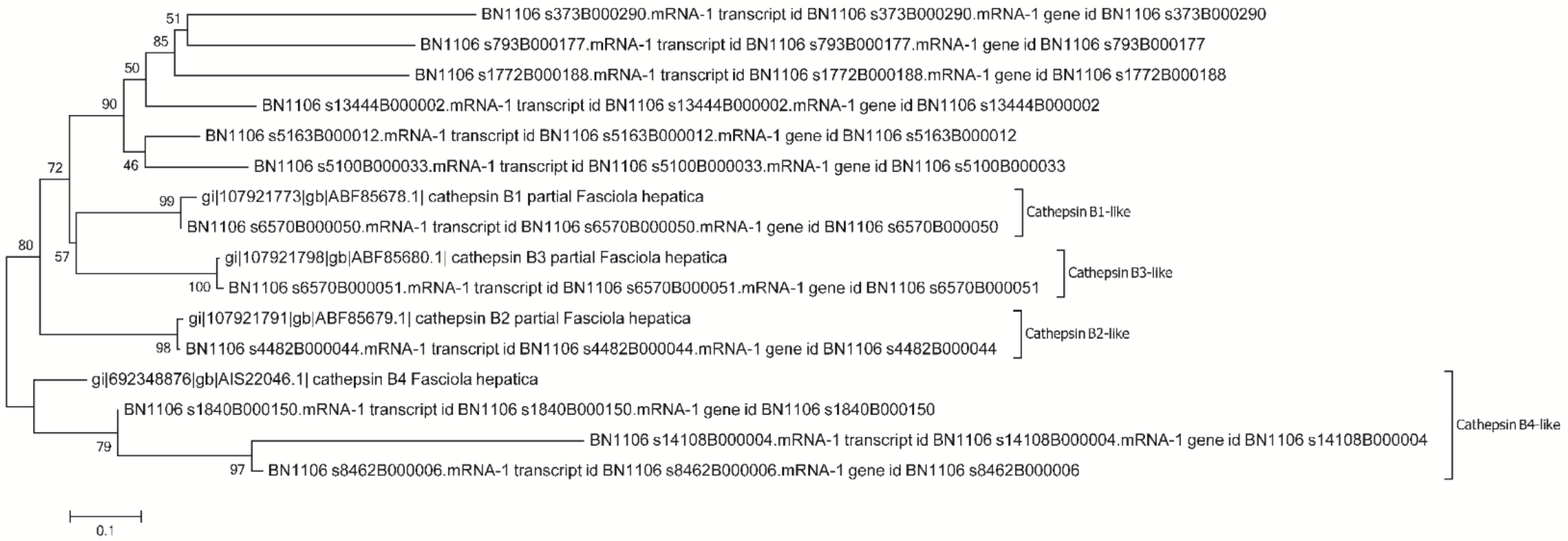

Supplementary Fig. S1. Phylogenetic relationships of the cathepsin B family amino acid sequences presented here is a bootstrapped (1,000 replicates) neighbor-joining phylogenetic unrooted tree. The percentages in the nodes represent bootstrap values and branch lengths are proportional to distances.

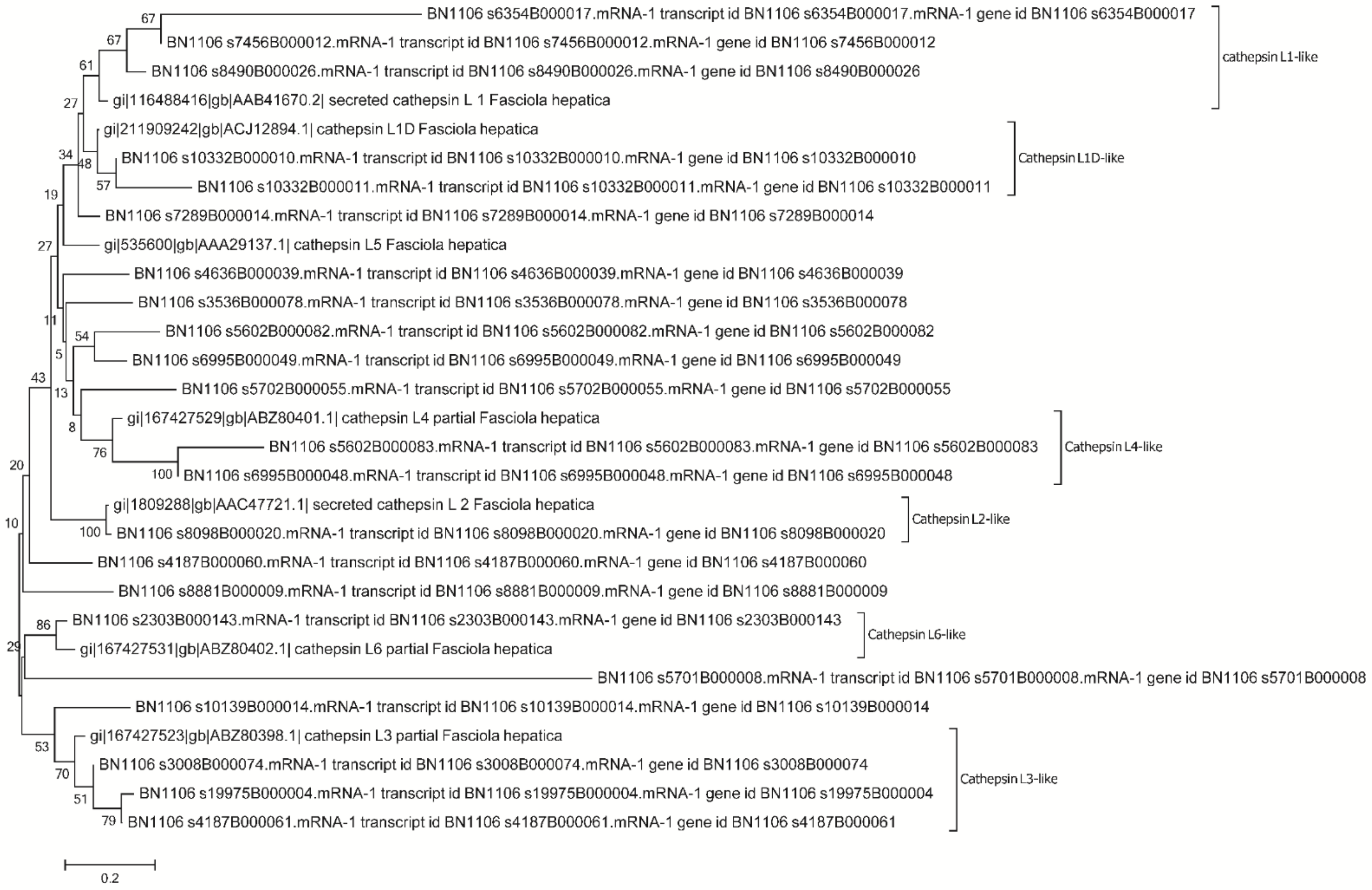

Supplementary Fig. S2. Phylogenetic relationships of the cathepsin L family amino acid sequences presented here is a bootstrapped (1,000 replicates) neighbor-joining phylogenetic unrooted tree. The percentages in the nodes represent bootstrap values and branch lengths are proportional to distances.

Supplementary Table S1. Differentially expressed proteins based on t-fold analyses comparing E/S products from NEJ stage with E/S products from the adult stage.

Accession number[a]	Description	Class	Fold Change[b]	p-value[b]	Regulation[c]	
					Adult E/S	NEJ E/S
BN1106_s6635B000017.mRNA-1	Legumain-1	Proteinase	5.78	0.00018		↑
BN1106_s9069B000006.mRNA-1	Legumain-1	Proteinase	7.77	0.01352		↑
BN1106_s5100B000033.mRNA-1	Cathepsin B-like	Proteinase	8.39	0.00645		↑
BN1106_s8462B000006.mRNA-1	Cathepsin B4 -like	Proteinase	8.40	0.01357		↑
BN1106_s6720B000015.mRNA-1	CD59-like protein	Immunity	9.67	0.00357		↑
BN1106_s2087B000065.mRNA-1	Legumain-1	Proteinase	9.88	0.00002		↑
BN1106_s5163B000012.mRNA-1	Cathepsin B-like	Proteinase	11.89	0.00026		↑
BN1106_s373B000290.mRNA-1	Cathepsin B-like	Proteinase	34.31	0.00390		↑
BN1106_s4651B000094.mRNA-1	Stefin-1	Proteinase inhibitor	35.86	0.00367	↑	
BN1106_s8881B000009.mRNA-1	Cathepsin L-like	Proteinase	48.74	0.00017		↑
BN1106_s3173B000376.mRNA-1	Alpha-glucosidase	Metabolism. carbohydrate	49.96	0.00947		↑

BN1106_s3008B000074.mRNA-1	Cathepsin L3-like	Proteinase	103.97	0.00349	↑
BN1106_s6570B000051.mRNA-1	Cathepsin B3-like	Proteinase	130.50	0.00002	↑
BN1106_s7612B000030.mRNA-1	Legumain	Proteinase	283.68	0.00044	↑

[a]Accession numbers of *Fasciola hepatica* proteins identified as differentially expressed (blue dots in Fig. 3B) (Cwiklinski et al., 2015a).

[b]Fold change and p-value of *Fasciola hepatica* proteins identified as differentially expressed (blue dots in Fig. 3B).

[c]Arrows are representing up-regulation (↑) of proteins identified as differentially in E/S products from NEJ stage or E/S products from the adult stage.

Supplementary Table S2. Differentially expressed proteins based on t-fold analyses comparing E/S products and somatic soluble proteins from NEJ stage.

Accession number[a]	Description	Class	Fold Change[b]	pValue[b]	Regulation[c]	
					NEJ E/S	Somatic NEJ
BN1106_s2907B000134	Actin-2	Cytoskeletal	-27.62	0.01065		↑
BN1106_s2907B000133	Actin-2	Cytoskeletal	-33.58	0.01004		↑
BN1106_s101B000531	Actin-2	Cytoskeletal	-45.75	0.00411		↑
BN1106_s455B000331	Actin-2	Cytoskeletal	-51.43	0.00058		↑
BN1106_s6570B000050	Cathepsin B1-like	Proteinase	4.78	0.00001	↑	
BN1106_s4482B000044	Cathepsin B2-like	Proteinase	5.82	0.00001	↑	
BN1106_s6570B000051	Cathepsin B3-like	Proteinase	6.00	0.00005	↑	
BN1106_s5163B000012	Cathepsin B-like	Proteinase	7.84	0.00023	↑	
BN1106_s5100B000033	Cathepsin B-like	Proteinase	7.40	0.00680	↑	
BN1106_s14108B000004	Cathepsin B-like	Proteinase	-1.68	0.01210		↑
BN1106_s3008B000074	Cathepsin L3-like	Proteinase	8.12	0.00086	↑	
BN1106_s4187B000061	Cathepsin L-like	Proteinase	9.32	0.00102	↑	
BN1106_s6720B000015	CD59-like protein	Immunity	4.56	0.00614	↑	
BN1106_s63B000399	CD59-like protein	Immunity	-10.06	0.00448		↑

BN1106_s7307B000022	Cubilin-like	Transport/storage	4.81	0.00001	↑	
BN1106_s17622B000002	Cubilin-like	Transport/storage	8.66	0.00057	↑	
BN1106_s1444B000095	Dynein	Cytoskeletal	-9.17	0.00271		↑
BN1106_s3227B000227	Enolase	Metabolism. carbohydrate	-10.22	0.00016		↑
tr|F2Z4I6|F2Z4I6_BOVIN	Histone H2A	Nuclear regulation	-3.90	0.01352		↑
tr|Q17QG8|Q17QG8_BOVIN	Histone H2A	Nuclear regulation	-7.21	0.00003		↑
BN1106_s7612B000030	Legumain	Proteinase	7.57	0.00007	↑	
BN1106_s7612B000031	Legumain	Proteinase	3.12	0.00075	↑	
BN1106_s2087B000065	Legumain-1	Proteinase	3.00	0.00001	↑	
BN1106_s6635B000017	Legumain-1	Proteinase	2.62	0.00009	↑	
BN1106_s10890B000012	Peptidase inhibitor 16	Cysteine-rich proteins	2.72	0.00002	↑	
BN1106_s5591B000098	Peptidase inhibitor 16	Cysteine-rich proteins	1.97	0.00401	↑	
BN1106_s246B000252	Phosphoenolpyruvate carboxykinase	Metabolism. carbohydrate	-24.20	0.01114		↑
BN1106_s1614B000280	Thioredoxin peroxidase	Oxidant metabolism/ detoxification	-10.72	0.00143		↑
BN1106_s5172B000090	Unknown product	Unknown	5.47	0.00238	↑	

Supplementary Table S3. *Fasciola hepatica*-derived proteins identified by LC-MS/MS. Protein functional classification and data generated in Bird's eye view from PatternLab for Proteomics platform (Carvalho et al., 2012a; Carvalho et al., 2012b) are provided in the accompanying spreadsheet (Hyperlinked Excel spreadsheet, zipped). Protein functional classification was manually curated using BLASTP searches against several databases. Accession number of *F. hepatica*-identified proteins, classification based in function and/or protein family, and accession numbers in addition to other parameters of best match identities obtained using BLASTP are provided ("Annotation" sheet). Data generated in the LC-MS/MS analyzes such as unique peptide, peptide count, spectral count, NSAF, and coverage are also provided for all triplicates from all three samples (in separated sheets).

Supplementary Table S4. *Bos taurus*-derived proteins identified by LC-MS/MS. Protein functional classification and data generated in Bird's eye view from PatternLab for Proteomics platform (Carvalho et al., 2012a; Carvalho et al., 2012b) are provided in the accompanying spreadsheet (Hyperlinked Excel spreadsheet, zipped). Protein functional classification was manually curated using BLASTP searches against several databases. Accession number of *F. hepatica*-identified proteins, classification based in function and/or protein family, and accession numbers in addition to other parameters of best match identities obtained using BLASTP are provided ("Annotation" sheet). Data generated in the LC-MS/MS analyzes such as unique peptide, peptide count, spectral count, NSAF, and coverage are also provided for all triplicates from all three samples (in separated sheets).

Supplementary Table S5. Differentially expressed proteins based on t-fold analyses comparing E/S products from NEJ stage with E/S products from the adult stage.

Accession number	Description	Class	Fold Change	pValue
BN1106_s2087B000065	Legumain-1	Secreted proteinase	-9.87	2.20E-05
BN1106_s3008B000074	Cathepsin L3-like	Secreted proteinase	-103.97	0.003495
BN1106_s3173B000376	Alpha-glucosidase	Carbohydrate metabolism	-49.96	0.009465
BN1106_s373B000290	Cathepsin B-like	Secreted proteinase	-34.31	0.003897
BN1106_s4651B000094	Stefin-1	Secreted proteinase inhibitor	35.86	0.003671
BN1106_s5100B000033	Cathepsin B-like	Secreted proteinase	-8.39	0.006446
BN1106_s5163B000012	Cathepsin B-like	Secreted proteinase	-11.88	0.000255
BN1106_s6570B000051	Cathepsin B3-like	Secreted proteinase	-130.49	2.49E-05
BN1106_s6635B000017	Legumain-1	Secreted proteinase	-5.78	0.000183
BN1106_s6720B000015	CD59-like protein	Immunity	-9.66	0.003575
BN1106_s7612B000030	Legumain	Secreted proteinase	-283.67	0.000443
BN1106_s8462B000006	Cathepsin B4 -like	Secreted proteinase	-8.40	0.013572
BN1106_s8881B000009	Cathepsin L-like	Secreted proteinase	-48.74	0.000167
BN1106_s9069B000006	Legumain-1	Secreted proteinase	-7.76	0.013517
tr|G3N0V2|G3N0V2_BOVIN	Keratin	Cytoskeletal	-20.71	0.017377
sp|P0CH28|UBC_BOVIN	Polyubiquitin-C	Proteasome machinery	7.98	0.021241
tr|Q17QG8|Q17QG8_BOVIN	Histone H2A	Nuclear regulation	-10.36	0.024623
BN1106_s8098B000020	Cathepsin L2-like	Secreted proteinase	34.91	0.026001
BN1106_s3227B000227	Enolase	Carbohydrate metabolism	-4.62	0.033486
BN1106_s7353B000023	Niemann-Pick protein	Lipid metabolism	6.84	0.037116
BN1106_s4026B000080	Thioredoxin	Oxidant metabolismo / detoxification	4.40	0.038137
BN1106_s4187B000060	Cathepsin L3-like	Secreted proteinase	-19.85	0.040523
tr|M0QVY0|M0QVY0_BOVIN	Keratin	Cytoskeletal	-14.63	0.042002
tr|F1MUY2|F1MUY2_BOVIN	Keratin	Cytoskeletal	-14.63	0.042002
BN1106_s6840B000044	Polyubiquitin-A	Proteasome machinery	16.76	0.045865
BN1106_s1110B000106	Cubilin	Transport/Storage	-7.22	0.058307

BN1106_s101B000531	Actin-2	Cytoskeletal	1.32	0.278262
BN1106_s114B000615	Cubilin-like	Transport/Storage	-1.91	0.080549
BN1106_s1444B000095	Dynein	Cytoskeletal	-1.35	0.277081
BN1106_s1614B000280	Thioredoxin peroxidase	Oxidant metabolismo/detoxification	4.13	0.031541
BN1106_s1657B000161	Tetraspanin-CD63 receptor	Signal transduction	-1.35	0.247136
BN1106_s1840B000150	Cathepsin B4-like	Secreted proteinase	-3.04	0.042116
BN1106_s1985B000403	Ectonucleotide pyrophosphatase / phosphodiesterase	Nuclear regulation	-4.62	0.058664
BN1106_s246B000252	Phosphoenolpyruvate carboxykinase (GTP)	Carbohydrate metabolism	-2.40	0.083656
BN1106_s2907B000133	Actin-2	Cytoskeletal	1.32	0.31012
BN1106_s3001B000132	Cubilin-like	Transport/Storage	-1.65	0.071483
BN1106_s3001B000132	Cubilin-like	Transport/Storage	-3.82	0.043215
BN1106_s3009B000044	Carbonic anhydrase 5B	Oxidant metabolismo/detoxification	1.68	0.259563
BN1106_s318B000274	Kunitz-type proteinase inhibitor	Secreted proteinase inhibitor	1.85	0.053469
BN1106_s4223B000091	Legumain-1	Secreted proteinase	1.24	0.301441
BN1106_s455B000331	Actin-2	Cytoskeletal	1.32	0.278262
BN1106_s4986B000028	SAP-1	Lipid metabolism	-2.68	0.053645
BN1106_s5246B000010	CD59-like protein	Immunity	-2.12	0.051046
BN1106_s5602B000082	Cathepsin L-like	Secreted proteinase	3.52	0.028177
BN1106_s5689B000026	Natterin-4	Lipid metabolism	-3.91	0.053573
BN1106_s584B000346	Glucose transporter-2 protein	Transport/Storage	3.02	0.231718
BN1106_s584B000350	Glucose transporter-2 protein	Transport/Storage	4.84	0.119918
BN1106_s617B000566	Leucine amino peptidase 1 C-terminal fragment	Secreted proteinase	1.35	0.217515
BN1106_s63B000399	CD59-like protein	Immunity	-1.06	4.19E-05
BN1106_s6995B000048	Cathepsin L4-like	Secreted proteinase	-2.89	0.117231

BN1106_s6995B000049	Cathepsin L-like	Secreted proteinase	-1.83	0.09822
BN1106_s7443B000031	Unknown product	Unknown	-2.47	0.064674
BN1106_s8038B000016	Unknown product	Unknown	3.04	0.092148
BN1106_s8826B000029	Kunitz-type proteinase inhibitor	Secreted proteinase inhibitor	1.98	0.060199
BN1106_s945B000218	Annexin A11	Cytoskeletal	2.64	0.050399
BN1106_s9797B000034	Leukotriene-A4 hydrolase	Transport/Storage	-2.77	0.015177
sp|P00760|TRY1_BOVIN	Cationic trypsin	Secreted proteinase	-2.21	0.037756
BN1106_s3001B000131	Cubilin-like	Transport/Storage	-3.41	0.001893

Each differently expressed protein is mapped according to its fold change and t-test p-value. Proteins are represented by: (blue shading) if had an identification that satisfied both fold and statistical criteria; (yellow shading) had an identifications that was filtered out by the L-stringency; (green shading) had an identification satisfied the fold criteria but, most likely, this happened by chance; and (red shading) had identification did not meet the fold and p-value criteria.

Supplementary Table S6. Differentially expressed proteins based on t-fold analyses comparing E/S products and somatic soluble NEJ.

Accession number	Description	Class	Fold Change	pValue
BN1106_s101B000531	actin-2	Cytoskeletal	-45.76	0.004111344
BN1106_s10890B000012	Cysteine-rich secretory protein family (GLIPR1)	Cysteine-rich proteins	2.73	1.71E-05
BN1106_s14108B000004	Cathepsin B-like	Secreted proteinase	-1.68	0.01209948
BN1106_s1444B000095	dynein	Cytoskeletal	-9.18	0.002711112
BN1106_s1614B000280	Thioredoxin peroxidase	Oxidant metabolismo/detoxification	-10.72	0.001428203
BN1106_s17622B000002	cubilin-like isoform 1	Transport/Storage	8.66	0.000574028
BN1106_s2087B000065	Legumain-1	Secreted proteinase	3.00	1.17E-05
BN1106_s246B000252	Phosphoenolpyruvate carboxykinase (GTP)	Carbohydrate metabolism	-24.21	0.01114098
BN1106_s2907B000133	actin-2	Cytoskeletal	-33.58	0.010035332
BN1106_s2907B000134	actin-2	Cytoskeletal	-27.63	0.010648377
BN1106_s3008B000074	s/protease	Secreted proteinase	8.13	0.000863597
BN1106_s3227B000227	enolase	Carbohydrate metabolism	-10.22	0.000162913
BN1106_s4187B000061	Cathepsin L-like	Secreted proteinase	9.33	0.00101565
BN1106_s4482B000044	Cathepsin B2-like	Secreted proteinase	5.82	1.13E-05
BN1106_s455B000331	actin-2	Cytoskeletal	-51.43	0.000582962
BN1106_s5100B000033	Cathepsin B-like	Secreted proteinase	7.41	0.006798594
BN1106_s5163B000012	Cathepsin B-like	Secreted proteinase	7.85	0.000226302
BN1106_s5172B000090	Unknown product	Unknown	5.48	0.002381086
BN1106_s5591B000098	CRISP3: cysteine-rich secretory protein	Cysteine-rich proteins	1.97	0.004006453
BN1106_s63B000399	CD59-like protein	Immunity	-10.06	0.004475068
BN1106_s6570B000050	Cathepsin B1-like	Secreted proteinase	4.78	1.00E-05
BN1106_s6570B000051	Cathepsin B3-like	Secreted proteinase	6.00	5.19E-05
BN1106_s6635B000017	Legumain-1	Secreted proteinase	2.63	9.30E-05
BN1106_s6720B000015	CD59-like protein	Immunity	4.56	0.006139616

BN1106_s7307B000022	cubilin	Transport/Storage	4.81	1.00E-05
BN1106_s7612B000030	Legumain	Secreted proteinase	7.58	7.01E-05
BN1106_s7612B000031	Legumain	Secreted proteinase	3.12	0.000752774
tr|F2Z4I6|F2Z4I6_BOVIN	Histone H2A	Nuclear regulation	-3.90	0.013522408
tr|Q17QG8|Q17QG8_BOVIN	Histone H2A	Nuclear regulation	-7.21	3.25E-05

BN1106_s4026B000080	Thioredoxin	Oxidant metabolismo/detoxification	-2.80	0.014941729
BN1106_s9797B000034	Leukotriene-A4 hydrolase - M1	Transport/Storage	3.79	0.016747702
BN1106_s4651B000094	Stefin-1	Secreted proteinase inhibitor	-3.39	0.017454129
BN1106_s26B000447	Collagen alpha-1(XV) chain	Extracellular matrix	-1.80	0.019348208
BN1106_s4187B000060	Cathepsin L3-like	Secreted proteinase	6.09	0.021285821
BN1106_s8462B000006	Cathepsin B4 -like	Secreted proteinase	3.34	0.026458637
sp|Q29S21|K2C7_BOVIN	Keratin type II cytoskeletal 7	Cytoskeletal	2.80	0.028125793
BN1106_s1840B000150	Cathepsin B4-like	Secreted proteinase	4.08	0.028955455
BN1106_s9069B000006	legumain-1	Secreted proteinase	1.91	0.029073677
BN1106_s9189B000015	peptidyl-prolyl cis-trans isomerase	Protein modification machinery	-1.58	0.030818119
sp|P0CH28|UBC_BOVIN	Polyubiquitin-C - cytoplasm - nucleus	Proteasome machinery	-1.65	0.032702869
BN1106_s3173B000376	alpha-glucosidase	Carbohydrate metabolism	11.48	0.035348373
BN1106_s10139B000014	Cathepsin L-like	Secreted proteinase	2.86	0.036243144
BN1106_s5602B000083	Cathepsin L4-like	Secreted proteinase	2.91	0.038707409
BN1106_s1985B000403	Ectonucleotide pyrophosphatase/phosphodiesterase	Nuclear regulation	10.49	0.043132688
BN1106_s114B000615	Cubilin-like	Transport/Storage	2.73	0.0433593
BN1106_s666B000200	mannosidase, alpha, class 2B	Carbohydrate metabolism	2.94	0.043498134
BN1106_s6995B000049	Cathepsin L-like	Secreted proteinase	7.03	0.043554602
BN1106_s6995B000048	Cathepsin L4-like	Secreted proteinase	4.14	0.045332674
BN1106_s373B000290	Cathepsin B-like	Secreted proteinase	1.60	0.056035137
BN1106_s4B000834	calcium-binding protein	Signal transduction	-2.20	0.063554229
BN1106_s4986B000028	SAP-1	Nuclear regulation	2.28	0.067300282
BN1106_s7443B000031	Unknown product	Unknown	2.32	0.071044603

BN1106_s2303B000143	Cathepsin L6-like	Secreted proteinase	4.83	0.098811878
BN1106_s7353B000023	Niemann-Pick protein	Lipid metabolism	-1.66	0.127000635
tr|F1MUY2|F1MUY2_BOVIN	keratin	Cytoskeletal	1.69	0.183014303
BN1106_s8038B000016	Unknown product	Unknown	1.38	0.053544143
BN1106_s3001B000132	cubilin-like	Transport/Storage	-1.22	0.069693987
BN1106_s3001B000132	cubilin-like	Transport/Storage	1.38	0.121027116
BN1106_s584B000346	Glucose transporter-2 protein	Transport/Storage	1.46	0.154081202
BN1106_s584B000350	Glucose transporter-2 protein	Transport/Storage	1.46	0.154081202
tr|F1MC11|F1MC11_BOVIN	keratin	Cytoskeletal	-1.41	0.163372128
tr|G3N0V2|G3N0V2_BOVIN	keratin	Cytoskeletal	1.32	0.167958883
sp|P00760|TRY1_BOVIN	Cationic trypsin	Secreted proteinase	1.27	0.172745702
BN1106_s6840B000044	Polyubiquitin-A	Proteasome machinery	-1.19	0.179563896
BN1106_s5689B000026	Natterin-4	Lipid metabolism	1.54	0.193585128
BN1106_s925B000547	Tubulin alpha-3	Cytoskeletal	-1.25	0.199430341
BN1106_s1110B000106	cubilin-like	Transport/Storage	1.39	0.20574462
BN1106_s318B000274	kunitz-type proteinase inhibitor	Secreted proteinase inhibitor	1.58	0.207665857
tr|M0QVY0|M0QVY0_BOVIN	keratin	Cytoskeletal	1.32	0.236544944
BN1106_s7273B000042	Major vault protein	Signal transduction	-7.10	8.72E-05
BN1106_s1200B000196	Unknown product	Unknown	2.11	0.000726494
BN1106_s617B000566	Leucine amino peptidase 1 fragment C-terminal - M17	Secreted proteinase	-6.77	0.001202213
BN1106_s1657B000161	Tetraspanin-CD63 receptor	Signal transduction	6.64	0.002766104
tr|F1MFW9|F1MFW9_BOVIN	keratin	Cytoskeletal	1.85	0.002858627
BN1106_s945B000218	Annexin A11	Cytoskeletal	-7.65	0.003350674
BN1106_s3001B000131	cubilin-like	Transport/Storage	-2.70	0.003584895
tr|E1BJB1|E1BJB1_BOVIN	The tubulin superfamily includes five distinct families	Cytoskeletal	-6.40	0.00531316
sp|P06394|K1C10_BOVIN	keratin	Cytoskeletal	-2.06	0.005752445
BN1106_s2275B000114	Uncharacterized protein	Unknown	1.82	0.009403358
BN1106_s114B000614	Cubilin	Transport/Storage	-3.86	0.010447237

Supplementary Table S7. Total protein comparison between Fh E/S products studies. [a]Robinson, M.W., Menon, R., Donnelly, S.M., Dalton, J.P., Ranganathan, S., 2009. An integrated transcriptomics and proteomics analysis of the secretome of the helminth pathogen Fasciola hepatica: proteins associated with invasion and infection of the mammalian host. Molecular & cellular proteomics : MCP 8, 1891-1907. [b]Hernandez-Gonzalez, A., Valero, M.L., del Pino, M.S., Oleaga, A., Siles-Lucas, M., 2010. Proteomic analysis of in vitro newly excysted juveniles from Fasciola hepatica. Molecular and biochemical parasitology 172, 121-128. [c]Wilson, R.A., Wright, J.M., de Castro-Borges, W., Parker-Manuel, S.J., Dowle, A.A., Ashton, P.D., Young, N.D., Gasser, R.B., Spithill, T.W., 2011. Exploring the Fasciola hepatica tegument proteome. International journal for parasitology 41, 1347-1359. [d]Cwiklinski, K., de la Torre Escudero, E., Trelis, M., Bernal, D., Dufresne, P.J., Brennan, G.P., O'Neill, S., Tort, J., Paterson, S., Marcilla, A., Dalton, J.P., Robinson, M.W., 2015b. The extracellular vesicles of the helminth pathogen, Fasciola hepatica: biogenesis pathways and cargo molecules involved in parasite pathogenesis. Molecular & cellular proteomics : MCP

Accession number	Seq name	Description	Class	This study			Robinson et al. 2008[a]		Hernández-González et al. 2010[b] Somatic NEJ	Wilson et al. 2011[c] Adult E/S products	Cwiklinski et al. 2015[d] Adult E/S products	Cwiklinski et al. 2015[d] Adult exosome proteins
				**E/S products NEJ**	**E/S products adult**	**Somatic soluble NEJ proteins**	**NEJ E/S products**	**Adult E/S products**				
**BN1106_s709B 000627**	114	ferritin	heme/iron related proteins		*						*	*
**BN1106_s1002 B000239**	130	ferritin	heme/iron related proteins			*						
**BN1106_s2101 B000084**	342	MF6p/FhH DM-1 protein	heme/iron related proteins		*	*	HAN4019c1 2.q1kT3				*	
**BN1106_s284B 000287**	439	myoglobin 1	heme/iron related proteins		*					FhC00255		*
**BN1106_s284B 000288**	440	myoglobin 1	heme/iron related proteins			*				FhB00042	*	
**BN1106_s3950 B000041**	564	ferritin	heme/iron related proteins		*	*					*	*

**BN1106_s917B000270**	876	ferritin	heme/iron related proteins			*		
**BN1106_s92B000564**	879	ferritin	heme/iron related proteins		*			
**BN1106_s101B000531**	2	Actin-2	Cytoskeletal	*	*	*	gi|1703101	
**BN1106_s103B000718**	5	Ankyrin 2b	Cytoskeletal			*		
**BN1106_s2349B000188**	48	Severin	Cytoskeletal			*		*
**BN1106_s2374B000246**	49	PDZ and LIM domain protein	Cytoskeletal			*		
**BN1106_s2906B000293**	60	lamin-C	Cytoskeletal			*		
**BN1106_s3147B000076**	66	dynein	Cytoskeletal			*		
**BN1106_s3353B000056**	69	synaptotagmin	Cytoskeletal		*			
**BN1106_s551B000321**	99	myosin	Cytoskeletal		*	*		*
**BN1106_s71B000363**	115	Ankyrin-2	Cytoskeletal			*		
**BN1106_s98B000745**	129	Titin	Cytoskeletal			*		
**BN1106_s103B000723**	138	Ankyrin	Cytoskeletal			*		
**BN1106_s1037B000175**	144	Na(+)/H(+) exchange regulatory cofactor NHE-RF1-like	Cytoskeletal			*		
**BN1106_s1042B000321**	145	thymosin	Cytoskeletal			*		

**BN1106_s1081 B000248**	156	Actin depolymerizing factor-like protein	Cytoskeletal			*			
**BN1106_s1096 B000199**	160	ankyrin repeat domain-containing protein	Cytoskeletal			*			
**BN1106_s1106 B000091**	163	Myosin-1	Cytoskeletal			*			
**BN1106_s1109 B000181**	164	Tubulin polymerization-promoting protein	Cytoskeletal			*			
**BN1106_s1111 B000208**	166	Microtubule-associated protein 1S	Cytoskeletal			*			
**BN1106_s1119 B000202**	169	Titin	Cytoskeletal			*			
**BN1106_s1168 B000108**	186	LIM domains protein	Cytoskeletal			*			
**BN1106_s1300 B000145**	213	moesin/ezrin/radixin homolog 1-like isoform X1	Cytoskeletal		*	*			*
**BN1106_s1403 B000129**	229	Plastin-2	Cytoskeletal		*	*	Fhep27d03.q1k	FhB02827	*
**BN1106_s1444 B000095**	235	dynein	Cytoskeletal	*	*	*			
**BN1106_s149B 000360**	238	Talin-1	Cytoskeletal			*			
**BN1106_s1515 B000336**	244	Filamin	Cytoskeletal			*			
**BN1106_s1582 B000145**	254	Dynein	Cytoskeletal		*	*			

**BN1106_s1582B000149**	255	Dynein	Cytoskeletal	*		
**BN1106_s19B000337**	311	Tropomodulin	Cytoskeletal	*		
**BN1106_s1922B000122**	315	paramyosin	Cytoskeletal	*	gi|126116628	
**BN1106_s1972B000196**	321	Tropomyosin	Cytoskeletal	*		
**BN1106_s2003B000172**	328	Cofilin	Cytoskeletal	*		
**BN1106_s2014B000189**	329	Nascent polypeptide-associated complex subunit alpha	Cytoskeletal	*		
**BN1106_s2018B000301**	330	Myosin-2	Cytoskeletal	*		FhC00899
**BN1106_s2132B000163**	347	adducin	Cytoskeletal	*		
**BN1106_s214B000743**	351	6 kDa tegumental protein	Cytoskeletal	*		
**BN1106_s2153B000115**	357	cofilin/tropomyosin type actin binding domain-containing protein	Cytoskeletal	*		
**BN1106_s227B000509**	370	Protein hu-li tai	Cytoskeletal	*		
**BN1106_s2291B000314**	376	Dynein	Cytoskeletal	*		
**BN1106_s2329B000134**	382	Actin related protein	Cytoskeletal	*		
**BN1106_s2333B000156**	384	Troponin I 4	Cytoskeletal	*		

**BN1106_s2349B000191**	385	Severin	Cytoskeletal			*			
**BN1106_s2434B000197**	397	Actin-interacting protein 1	Cytoskeletal			*			
**BN1106_s2590B000129**	412	PDZ and LIM domain protein Zasp	Cytoskeletal			*			
**BN1106_s2864B000144**	445	Dynein	Cytoskeletal			*			
**BN1106_s2907B000132**	451	actin-2	Cytoskeletal			*			
**BN1106_s2907B000133**	452	actin-2	Cytoskeletal	*	*	*			*
**BN1106_s2907B000134**	453	actin-2	Cytoskeletal	*		*			
**BN1106_s2949B000195**	457	Microtubule-associated protein RP/EB family member 1	Cytoskeletal			*			
**BN1106_s296B000186**	458	Filamin	Cytoskeletal			*		*	
**BN1106_s3045B000180**	470	Troponin T	Cytoskeletal			*			
**BN1106_s3182B000117**	488	Myosin motor domain	Cytoskeletal			*			
**BN1106_s322B000089**	491	Titin	Cytoskeletal			*			
**BN1106_s3225B000128**	492	coronin	Cytoskeletal			*			
**BN1106_s323B000257**	495	Myosin motor domain	Cytoskeletal			*			
**BN1106_s323B000258**	496	Myosin tail	Cytoskeletal			*	gi|161044/gi|76154815		

**BN1106_s3266 B000046**	499	Annexin	Cytoskele tal		*				*
**BN1106_s3478 B000064**	523	alpha- actinin	Cytoskele tal			*			
**BN1106_s3509 B000140**	524	Microtubule -associated protein 1	Cytoskele tal			*			
**BN1106_s392B 000871**	558	Tubulin beta-2C chain	Cytoskele tal			*			
**BN1106_s4069 B000247**	578	alpha actinin	Cytoskele tal			*			*
**BN1106_s410B 000441**	582	Titin	Cytoskele tal			*			
**BN1106_s410B 000444**	583	Titin	Cytoskele tal			*			
**BN1106_s410B 000448**	585	Titin	Cytoskele tal			*			
**BN1106_s4130 B000080**	587	Tropomyosi n-2	Cytoskele tal			*			
**BN1106_s4255 B000066**	600	spectrin alpha	Cytoskele tal			*			
**BN1106_s455B 000331**	632	actin-2	Cytoskele tal	*	*	*	gi|1703114	FhB00085	
**BN1106_s476B 000184**	650	adducin	Cytoskele tal			*			
**BN1106_s500B 000161**	668	Annexin A6	Cytoskele tal		*	*			*
**BN1106_s502B 000344**	671	CG34417 - actin binding	Cytoskele tal			*			
**BN1106_s5179 B000059**	682	Tubulin- specific chaperone A	Cytoskele tal			*			
**BN1106_s527B 000393**	689	Myosin	Cytoskele tal			*	gi|262213552		
**BN1106_s5331 B000045**	694	LIM and SH3 domain protein	Cytoskele tal			*			

**BN1106_s55B000372**	701	tubulin	Cytoskeletal			*				*
**BN1106_s567B000346**	717	Profilin	Cytoskeletal			*				
**BN1106_s586B000372**	736	Cofilin	Cytoskeletal			*				
**BN1106_s602B000099**	744	tubulin polymerization-promoting protein family member	Cytoskeletal			*				
**BN1106_s647B000405**	767	Tropomyosin	Cytoskeletal			*	gi|29337029			
**BN1106_s656B000153**	771	Laminin	Cytoskeletal			*				
**BN1106_s727B000100**	798	LIM domains protein 3	Cytoskeletal			*				
**BN1106_s819B000364**	848	Annexin A13	Cytoskeletal		*	*		FhB01618/ FhB02550	*	*
**BN1106_s819B000365**	849	Annexin A3	Cytoskeletal			*				
**BN1106_s90B000601**	868	LIM domains protein 2	Cytoskeletal			*				
**BN1106_s925B000539**	881	Tubulin	Cytoskeletal			*				
**BN1106_s925B000543**	882	Tubulin	Cytoskeletal			*	gi|195157178			
**BN1106_s925B000547**	883	Tubulin alpha-3	Cytoskeletal	*		*				
**BN1106_s937B000520**	890	Myosin	Cytoskeletal			*				
**BN1106_s945B000218**	894	Annexin A11	Cytoskeletal	*	*	*		FhB01398	*	*
**BN1106_s949B000142**	899	dynein light chain	Cytoskeletal		*	*				

**BN1106_s949B000146**	900	Dynein light chain LC8	Cytoskeletal		*	*						
**BN1106_s10058B000012**	1	Hydroxyglutarate dehydrogenase	Oxidant metabolismo/detoxification			*						
**BN1106_s3005B000095**	64	Thioredoxin-glutathione reductase	Oxidant metabolismo/detoxification		*	*				gi|157887771		
**BN1106_s638B000318**	106	glutathione dehydrogenase	Oxidant metabolismo/detoxification			*						
**BN1106_s1029B000154**	137	glutathione S-transferase	Oxidant metabolismo/detoxification			*						
**BN1106_s10348B000022**	143	Glutathione peroxidase	Oxidant metabolismo/detoxification		*							
**BN1106_s1061B000223**	151	glutaredoxin	Oxidant metabolismo/detoxification			*						
**BN1106_s1081B000242**	155	Glutathione S-transferase	Oxidant metabolismo/detoxification		*	*					*	*
**BN1106_s1459B000183**	236	Malate dehydrogenase	Oxidant metabolismo/detoxification		*	*						*
**BN1106_s1614B000280**	261	Thioredoxin peroxidase	Oxidant metabolismo/detoxification	*	*	*				FhB00158	*	*

**BN1106_s2277 B000049**	374	Retinol dehydrogenase 12	Oxidant metabolismo/detoxification		*	*			*
**BN1106_s2507 B000140**	407	Retinol dehydrogenase 12	Oxidant metabolismo/detoxification		*				
**BN1106_s2745 B000189**	429	Glyoxalase domain-containing protein	Oxidant metabolismo/detoxification			*			
**BN1106_s2937 B000224.mRNA-1 transcript_id=BN1106_s2937B000224.mRNA-1 gene_id=BN1106_s2937B000224**	456	Transketolase	Oxidant metabolismo/detoxification			*			
**BN1106_s3009 B000044**	465	Carbonic anhydrase 5B	Oxidant metabolismo/detoxification	*	*		FhB00925		*
**BN1106_s3189 B000243**	489	Superoxide dismutase Cu-Zn	Oxidant metabolismo/detoxification		*	*			
**BN1106_s3595 B000059**	536	Glutathione S-transferase class-mu 26 kDa isozyme 1	Oxidant metabolismo/detoxification			*		*	

**BN1106_s3715B000086**	546	carbonyl reductase	Oxidant metabolismo/detoxification			*						
**BN1106_s3834B000075**	552	isocitrate dehydrogenase 2 (NADP+)	Oxidant metabolismo/detoxification			*						
**BN1106_s3875B000041**	555	carbonic anhydrase II	Oxidant metabolismo/detoxification			*						
**BN1106_s4026B000080**	574	Thioredoxin	Oxidant metabolismo/detoxification	*	*	*	HAN4018d09.q1kT3	Q9U1G7			*	*
**BN1106_s4027B000177**	575	alcohol dehydrogenase	Oxidant metabolismo/detoxification			*						
**BN1106_s4097B000030**	580	Malate dehydrogenase	Oxidant metabolismo/detoxification			*						
**BN1106_s4370B000168**	613	Glutathione S-transferase	Oxidant metabolismo/detoxification		*							
**BN1106_s444B000267**	618	Peroxiredoxin 3	Oxidant metabolismo/detoxification			*	P91883/HAN4004e01.q1kT3/HAN4005g04.q1kT3		gi|166235906			
**BN1106_s4479B000057**	623	Glutathione S-transferase	Oxidant metabolismo/detoxification		*	*				FhB00340	*	

**BN1106_s5500B000138**	703	Superoxide dismutase	Oxidant metabolismo/detoxification		*			
**BN1106_s5504B000045**	704	glutathione S-transferase	Oxidant metabolismo/detoxification		*			
**BN1106_s567B000345**	716	L-lactate dehydrogenase - it is released during tissue damage	Oxidant metabolismo/detoxification		*			
**BN1106_s645B000322**	765	Aldehyde dehydrogenase	Oxidant metabolismo/detoxification		*			
**BN1106_s7830B000018**	836	Glutathione S-transferase	Oxidant metabolismo/detoxification	*		gi|452903	*	
**BN1106_s9130B000051**	872	hydroxyacyl glutathione hydrolase	Oxidant metabolismo/detoxification		*			
**BN1106_s9271B000022**	884	Thioredoxin-glutathione reductase	Oxidant metabolismo/detoxification	*	*		*	
**BN1106_s1191B000313**	12	Von Willebrand factor A domain-containing protein / Vacuolar	Oxidant metabolismo/detoxification	*				*

		protein sorting 26				
**BN1106_s115B 000510**	180	Tenascin	Oxidant metabolismo/detoxification		*	
**BN1106_s1246 B000440**	201	CRE-LEC-2 protein	Oxidant metabolismo/detoxification		*	
**BN1106_s176B 000277**	286	Collagen alpha-1(IV) chain	Oxidant metabolismo/detoxification		*	
**BN1106_s176B 000279**	287	Collagen alpha-1(IV) chain	Oxidant metabolismo/detoxification		*	*
**BN1106_s1922 B000120**	314	Periostin	Oxidant metabolismo/detoxification	*		*
**BN1106_s2057 B000129**	336	spectrin	Oxidant metabolismo/detoxification		*	
**BN1106_s2351 B000181**	386	spectrin beta chain	Oxidant metabolismo/detoxification		*	

**BN1106_s2354B000024**	387	Basement membrane-specific heparan sulfate proteoglycan core protein	Oxidant metabolismo/detoxification			*		*
**BN1106_s25B000189**	405	Basement membrane-specific heparan sulfate proteoglycan core protein	Oxidant metabolismo/detoxification			*		*
**BN1106_s26B000447**	414	Collagen alpha-1(XV) chain	Oxidant metabolismo/detoxification	*		*		
**BN1106_s392B000875**	559	ARM-1 protein	Oxidant metabolismo/detoxification			*		
**BN1106_s462B000766**	635	Fibropellin-1	Oxidant metabolismo/detoxification			*		*
**BN1106_s503B000225**	672	Innexin	Oxidant metabolismo/detoxification			*		
**BN1106_s6025B000064**	745	Von Willebrand factor A domain-containing protein	Oxidant metabolismo/detoxification		*		FhC01779	

**BN1106_s1442B000167**	234	CD59-like protein	Immunity		*			
**BN1106_s243B000419**	395	CD59-like protein	Immunity			*		
**BN1106_s5246B000010**	688	CD59-like protein	Immunity	*	*			
**BN1106_s63B000399**	760	CD59-like protein	Immunity	*	*	*		
**BN1106_s6720B000015**	778	CD59-like protein	Immunity	*	*	*		
**BN1106_s92B000559**	878	T-cell immunomodulatory protein	Immunity	*				
**BN1106_s2898B000141**	59	Cysteine synthase	Amino acid metabolism			*		
**BN1106_s1098B000219**	161	Pyrroline-5-carboxylate reductase	Amino acid metabolism			*		
**BN1106_s232B000326**	381	TyrA protein	Amino acid metabolism			*		
**BN1106_s2400B000186**	391	Glutamine synthase	Amino acid metabolism			*		*
**BN1106_s2851B000085**	443	Aspartate aminotransferase	Amino acid metabolism		*			
**BN1106_s398B000241**	567	Ornithine aminotransferase	Amino acid metabolism			*	*	

**BN1106_s4261B000116**	602	Taurocyamine kinase	Amino acid metabolism		*	
**BN1106_s436B000498**	610	Fumarate hydratase class I	Amino acid metabolism		*	
**BN1106_s4661B000179**	639	Glutamine synthetase	Amino acid metabolism		*	
**BN1106_s4661B000180**	640	Glutamine synthetase	Amino acid metabolism		*	
**BN1106_s5767B000030**	726	Glutamate dehydrogenase	Amino acid metabolism		*	*
**BN1106_s771B000469**	828	Alpha-aminoadipic semialdehyde synthase	Amino acid metabolism		*	
**BN1106_s1241B000260**	17	N-acetyl galactosaminidase	Carbohydrate metabolism		*	
**BN1106_s1959B000206**	35	Glucose-6-phosphate isomerase	Carbohydrate metabolism		*	
**BN1106_s2385B000108**	50	Lysosomal alpha-glucosidase	Carbohydrate metabolism	*		

**BN1106_s244B000349**	52	UTP-glucose-1-phosphate uridylyltransferase	Carbohydrate metabolism			*	
**BN1106_s3173B000376**	67	alpha-glucosidase	Carbohydrate metabolism	*	*	*	*
**BN1106_s3375B000064**	70	Fructose-1,6-bisphosphatase 1	Carbohydrate metabolism			*	
**BN1106_s531B000172**	97	dihydrolipoamide acetyltransferase component of pyruvate dehydrogenase	Carbohydrate metabolism			*	
**BN1106_s7879B000034**	120	Glucose-6-phosphate isomerase	Carbohydrate metabolism			*	
**BN1106_s8157B000032**	121	Dihydrolipoamide dehydrogenase	Carbohydrate metabolism			*	*
**BN1106_s1026B000549**	135	Aldose reductase	Carbohydrate metabolism			*	
**BN1106_s1298B000178**	212	Galactokinase-like protein	Carbohydrate metabolism			*	

**BN1106_s1518B000071**	245	Fructose-bisphosphate aldolase	Carbohydrate metabolism			*					
**BN1106_s1551B000468**	248	Propionyl-CoA carboxylase beta chain	Carbohydrate metabolism			*					*
**BN1106_s1672B000086**	271	Pyruvate kinase	Carbohydrate metabolism			*					
**BN1106_s175B000200**	283	Hexokinase	Carbohydrate metabolism		*	*			FhB00376	*	*
**BN1106_s219B000273**	363	1,4-alpha-glucan-branching enzyme	Carbohydrate metabolism			*					
**BN1106_s246B000252**	399	Phosphoenolpyruvate carboxykinase (GTP)	Carbohydrate metabolism	*	*	*	Fhep07g02.q1k	gi|167541044	FhB01525		
**BN1106_s269B000233**	422	Phosphoglucomutase-1	Carbohydrate metabolism			*					
**BN1106_s3213B000041**	490	Triosephosphate isomerase	Carbohydrate metabolism		*	*			FhB00282	*	
**BN1106_s3227B000227**	494	enolase	Carbohydrate metabolism	*	*	*	Q27655.1/HAN4009g10.q1kT3	gi|3023708	FhB00588	*	*
**BN1106_s393B000274**	560	Phosphoglucomutase	Carbohydrate			*					

			metabolism									
**BN1106_s395B000721**	563	Phosphoenolpyruvate carboxykinase	Carbohydrate metabolism			*						
**BN1106_s4083B000032**	579	glycogen debranching enzyme-like isoform X2	Carbohydrate metabolism			*						
**BN1106_s4361B000046**	611	Pyruvate kinase	Carbohydrate metabolism			*						
**BN1106_s4469B000065**	622	aldolase A, fructose-bisphosphate	Carbohydrate metabolism			*	HAN4004c01.q1kT3		gi|1703248	FhB00113	*	*
**BN1106_s4504B000167**	626	Aldose 1-epimerase	Carbohydrate metabolism			*						
**BN1106_s561B000223**	713	ATP-dependent 6-phosphofructokinase	Carbohydrate metabolism			*						
**BN1106_s580B000238**	730	Glycogenin glucosyltransferase	Carbohydrate metabolism			*						
**BN1106_s666B000200**	777	mannosidase, alpha, class 2B	Carbohydrate metabolism	*		*						*
**BN1106_s8460B000014**	854	transaldolase 1	Carbohydrate			*						

			metabolism									
**BN1106_s85B000787**	856	Transaldolase	Carbohydrate metabolism			*						
**BN1106_s916B000192**	875	Alpha-1,4 glucan phosphorylase	Carbohydrate metabolism			*						
**BN1106_s233B000262**	47	NADP-dependent malic enzyme	Energy metabolism			*				FhB01995		*
**BN1106_s11911B000016**	191	Mitochondrial acetate:succinate	Energy metabolism			*						
**BN1106_s1501B000239**	241	Succinate dehydrogenase ubiquinone flavoprotein subunit	Energy metabolism			*			gi|195436412			
**BN1106_s1848B000328**	302	inorganic pyrophosphatase 1	Energy metabolism			*						
**BN1106_s1866B000129**	306	ATP synthase	Energy metabolism			*	HAN3004-1a05.q1k		gi|56758584			
**BN1106_s2001B000142**	326	Cytochrome c proximal	Energy metabolism			*						
**BN1106_s2574B000116**	410	acyl-CoA-binding protein (ACBP)/diazepam	Energy metabolism			*						

		binding inhibitor (DBI).						
**BN1106_s2896B000173**	449	NADH-cytochrome b5 reductase	Energy metabolism		*			
**BN1106_s3430B000064**	518	Succinate dehydrogenase	Energy metabolism		*			
**BN1106_s3452B000178**	520	methylmalonyl-CoA mutase - involved in key metabolic pathways	Energy metabolism		*			
**BN1106_s4332B000087**	608	F0F1 ATP synthase subunit alpha	Energy metabolism		*			*
**BN1106_s444B000268**	619	Citrate synthase	Energy metabolism		*			
**BN1106_s5004B000026**	669	succinyl-CoA ligase	Energy metabolism		*			
**BN1106_s5174B000030**	681	Glyceraldehyde-3-phosphate dehydrogenase - GAIT complex	Energy metabolism	*	*	Fhep15c10.q1k	gi|16406594	*
**BN1106_s6083B000078**	753	Succinyl-CoA ligase subunit beta	Energy metabolism		*			

**BN1106_s6797B000034**	781	Malate dehydrogenase	Energy metabolism		*					
**BN1106_s2194B000230**	364	Succinyl-CoA synthetase alpha subunit	Intermediate metabolism		*					
**BN1106_s1285B000159**	18	Acid sphingomyelinase-like phosphodiesterase	Lipid metabolism	*					*	*
**BN1106_s1498B000257**	26	Niemann-Pick C1	Lipid metabolism	*						*
**BN1106_s771B000467**	118	Fatty-acid amide hydrolase	Lipid metabolism	*						
**BN1106_s10326B000017.**	141	saposin-like (FhSAP-3)	Lipid metabolism	*					*	*
**BN1106_s1228B000120**	196	Fatty acid-binding protein type 2	Lipid metabolism	*	*	Q7M4G1/Fhep46d02.q1k/Fhep20f11.q1k	Q7M4G1		*	
**BN1106_s1252B000359**	203	Propionyl-CoA carboxylase	Lipid metabolism		*					*
**BN1106_s1597B000141**	258	LAMA-like protein 2	Lipid metabolism	*				FhB00284		
**BN1106_s1639B000396**	267	Prostamide/prostaglandin F synthase	Lipid metabolism		*					
**BN1106_s20469B000004**	334	Niemann-Pick type C2	Lipid metabolism	*						*

**BN1106_s2223 B000190**	366	Threonyl-tRNA synthetase	Lipid metabolism			*						
**BN1106_s2258 B000081**	369	Niemann-Pick protein	Lipid metabolism	*								
**BN1106_s2495 B000112**	404	Niemann-Pick type C2	Lipid metabolism		*							
**BN1106_s3703 B000103**	545	acetate:succinate CoA-transferase	Lipid metabolism			*						
**BN1106_s4047 B000060**	577	Glycerol-3-phosphate dehydrogenase	Lipid metabolism			*						
**BN1106_s4759 B000058**	649	Natterin-4	Lipid metabolism			*						
**BN1106_s4986 B000028**	665	saposin-like protein 1 (FhSAP1)	Lipid metabolism	*	*	*						
**BN1106_s4998 B000033**	666	Group XV phospholipase A2	Lipid metabolism		*					FhB00083	*	
**BN1106_s5103 B000076**	678	acetyl-CoA acetyltransferase 2	Lipid metabolism			*						
**BN1106_s5689 B000026**	721	Natterin-4	Lipid metabolism	*	*	*					*	*
**BN1106_s6908 B000039**	786	Niemann-Pick protein	Lipid metabolism		*							
**BN1106_s706B 000207**	792	methylmalonyl-CoA epimerase	Lipid metabolism			*						

**BN1106_s7353B000023**	803	Niemann-Pick protein	Lipid metabolism	*	*	*		*
**BN1106_s7521B000031**	821	Saposin-like protein 2 (FhSAP2)	Lipid metabolism		*			
**BN1106_s2044B000106**	333	purine nucleoside phosphorylase 5a - transferase	Nuclear metabolism			*		
**BN1106_s2970B000126**	460	Deoxyribonuclease	Nuclear metabolism			*		
**BN1106_s3026B000095**	466	5'-bisphosphate nucleotidase	Nuclear metabolism			*		
**BN1106_s375B000232**	550	Staphylococcal nuclease domain-containing protein 1	Nuclear metabolism			*		
**BN1106_s754B000176**	822	Adenosine deaminase - Cat eye syndrome critical region protein 5	Nuclear metabolism			*		
**BN1106_s780B000236**	834	adenylate kinase	Nuclear metabolism			*		
**BN1106_s992B000187**	905	APEX nuclease	Nuclear metabolism			*		
**BN1106_s6B000373**	741	arginine/serine-rich	ne			*		

		splicing factor							
**BN1106_s1454 B000184**	22	Histone H2A	Nuclear regulation	*				gi|51701468	
**BN1106_s1596 B000265**	29	Ectonucleotide pyrophosphatase/phosphodiesterase	Nuclear regulation	*					
**BN1106_s1789 B000241**	31	Barrier-to-autointegration factor	Nuclear regulation			*			
**BN1106_s1985 B000403**	38	Ectonucleotide pyrophosphatase/phosphodiesterase	Nuclear regulation	*	*	*			*
**BN1106_s3725 B000198**	76	Histone H2A	Nuclear regulation			*	HAN5021d04.q1kT3		
**BN1106_s557B 000139**	100	AHNAK-like	Nuclear regulation			*			
**BN1106_s6466 B000009**	107	Histone H3	Nuclear regulation			*		gi|2116601	
**BN1106_s674B 000165**	112	Histone H3	Nuclear regulation			*			
**BN1106_s7720 B000056**	119	dTMP kinase	Nuclear regulation			*			
**BN1106_s1641 B000184**	268	DNA-binding protein A	Nuclear regulation			*			
**BN1106_s1958 B000299**	319	High mobility group protein	Nuclear regulation			*			
**BN1106_s1987 B000231**	322	Cellular nucleic acid-binding protein	Nuclear regulation			*			

**BN1106_s2003B000165**	327	Histone H2A	Nuclear regulation			*				
**BN1106_s2127B000101**	346	DEK protein	Nuclear regulation			*				
**BN1106_s2195B000129**	365	Zinc finger RNA-binding protein	Nuclear regulation			*				
**BN1106_s2761B000168**	433	Uracil-DNA glycosylase	Nuclear regulation			*				
**BN1106_s306B000267**	471	Telomerase protein component 1	Nuclear regulation		*					FhC05121
**BN1106_s41B000298**	581	High mobility group protein	Nuclear regulation			*				
**BN1106_s5595B000080**	710	chromobox homolog 1 - histone methyltransferase binding	Nuclear regulation			*				
**BN1106_s596B000431**	740	UV excision repair protein RAD23	Nuclear regulation			*				
**BN1106_s8B000460**	843	Histone H1/5	Nuclear regulation			*				
**BN1106_s8822B000019**	863	Histone H2B	Nuclear regulation			*	HAN5013f06.q1kT3			
**BN1106_s107B000175**	7	fasciclin-2-like	Protein export machinery			*				

**BN1106_s418B000293**	85	Golgi-associated plant pathogenesis-related protein 1	Protein export machinery		*
**BN1106_s103B000726**	139	Synaptic vesicle membrane protein VAT-1	Protein export machinery		*
**BN1106_s103B000727**	140	Synaptic vesicle membrane protein VAT-1	Protein export machinery		*
**BN1106_s10435B000022**	147	Phosphoglucomutase-1	Protein export machinery		*
**BN1106_s1094B000139**	158	Stress-induced-phosphoprotein 1	Protein export machinery		*
**BN1106_s10981B000052**	162	NSF attachment protein SNAP	Protein export machinery		*
**BN1106_s1142B000130**	176	Syntaxin 1A	Protein export machinery	*	
**BN1106_s1956B000118**	318	Golgi-associated plant pathogenesis-related protein 1	Protein export machinery		*

**BN1106_s1995 B000318**	323	Fasciclin I-like protein	Protein export machinery	*			*	
**BN1106_s2505 B000147**	406	phosphogluconate dehydrogenase	Protein export machinery			*		
**BN1106_s3033 B000087**	468	phosphoglycerate kinase	Protein export machinery			*		
**BN1106_s3747 B000112**	549	ST1 homolog	Protein export machinery		*	*		*
**BN1106_s390B 000196**	556	Syntenin-1	Protein export machinery		*			
**BN1106_s449B 000179**	625	clathrin complex	Protein export machinery			*		
**BN1106_s5131 B000049**	679	coatomer protein complex	Protein export machinery			*		
**BN1106_s5333 B000045**	695	Lethal(2) giant larvae protein homolog 1	Protein export machinery			*		
**BN1106_s5369 B000082**	699	Transitional endoplasmic reticulum ATPase	Protein export machinery			*		
**BN1106_s7866 B000032**	837	phosphoglycerate mutase	Protein export			*		

			machinery					
**BN1106_s11916B000010**	13	DnaJ homolog subfamily	Protein modification machinery		*			
**BN1106_s1320B000236**	19	heat shock protein 83 kDa	Protein modification machinery		*		FhB00008	
**BN1106_s1679B000169**	30	universal stress protein	Protein modification machinery		*			
**BN1106_s2982B000132**	63	peptidylglycine alpha-hydroxylating monooxygenase	Protein modification machinery					
**BN1106_s309B000234**	65	heat shock 70 kDa protein	Protein modification machinery	*	*	gi|552242	FhB00129	*
**BN1106_s3937B000101.**	82	Molecular chaperone HtpG	Protein modification machinery		*			
**BN1106_s495B000230**	91	heat shock 70 kDa	Protein modification machinery		*			

**BN1106_s9461 B000006**	128	heat shock 70 kDa	Protein modification machinery		*	
**BN1106_s1057 B000126**	149	DnaJ homolog subfamily A member 1	Protein modification machinery	*	*	
**BN1106_s1147 B000259**	179	heat shock protein 70	Protein modification machinery		*	
**BN1106_s1242 B000159**	200	DNAJ homolog subfamily C member 11	Protein modification machinery		*	*
**BN1106_s1396 B000332**	224	Methyltransferase	Protein modification machinery		*	
**BN1106_s14B0 00365**	227	Protein archease - extracellular vesicular exosome	Protein modification machinery	*		
**BN1106_s1793 B000159**	293	chaperonin containing t-complex	Protein modification machinery		*	
**BN1106_s2018 B000302**	331	chaperonin containing Tcp1	Protein modification		*	

			machinery									
**BN1106_s2179 B000235**	362	heat shock factor-binding protein 1-like	Protein modification machinery			*						
**BN1106_s2415 B000155**	393	Acidic leucine-rich nuclear phosphoprotein 32 family member B	Protein modification machinery			*						
**BN1106_s2487 B000188**	403	DnaK-type molecular chaperone	Protein modification machinery			*						
**BN1106_s2641 B000139**	417	Aconitate hydratase 1	Protein modification machinery			*						
**BN1106_s2740 B000079**	427	HSP90	Protein modification machinery			*						
**BN1106_s2763 B000063**	434	Protein disulfide-isomerase	Protein modification machinery			*					*	
**BN1106_s2909 B000085**	454	10 kDa heat shock protein	Protein modification			*						

			machinery	
**BN1106_s332B000232**	505	Adenosylhomocysteinase	Protein modification machinery	*
**BN1106_s3324B000219**	507	chaperonin family - cpn60	Protein modification machinery	*
**BN1106_s3592B000072**	535	DnaJ (Hsp40) homolog	Protein modification machinery	*
**BN1106_s369B000200**	543	heat shock protein	Protein modification machinery	*
**BN1106_s3867B000081**	553	T-complex protein 1 subunit zeta - chaperonin containing Tcp1	Protein modification machinery	*
**BN1106_s4119B000141**	586	T-complex protein 1 subunit alpha	Protein modification machinery	*
**BN1106_s470B000292**	643	heat shock protein p36-like	Protein modification machinery	*

**BN1106_s4703B000071**	644	Chaperonin GroEL	Protein modification machinery	*
**BN1106_s4999B000041**	667	protein disulfide-isomerase A3-like	Protein modification machinery	*
**BN1106_s5284B000043**	691	Peptidyl-prolyl cis-trans isomerase B - therefore function as protein folding chaperones	Protein modification machinery	*
**BN1106_s5514B000140**	705	Chaperonin containing TCP1	Protein modification machinery	*
**BN1106_s553B000158**	706	Calnexin	Protein modification machinery	*
**BN1106_s5618B000057**	714	protein disulfide-isomerase A6-like	Protein modification machinery	*
**BN1106_s58B000487**	729	Degradation arginine-rich protein for mis-folding	Protein modification machinery	*

**BN1106_s6031B000032**	746	Alpha crystallin-containing small heat shock protein variant	Protein modification machinery		*	
**BN1106_s6241B000014**	757	Peptidyl-prolyl cis-trans isomerase	Protein modification machinery		*	
**BN1106_s639B000754**	764	isochorismatase domain containing 1	Protein modification machinery		*	
**BN1106_s6573B000067**	773	Peptidyl-prolyl cis-trans isomerase FKBP5	Protein modification machinery		*	
**BN1106_s746B000284**	819	Chaperonin containing TCP1, subunit 3	Protein modification machinery		*	
**BN1106_s7787B000022**	833	T-complex protein 1 subunit delta	Protein modification machinery		*	
**BN1106_s9189B000015**	877	peptidyl-prolyl cis-trans isomerase	Protein modification machinery	*	*	FhB03741

**BN1106_s208B000185**	39	ubiquitin-protein ligase BRE1	Proteosome machinery		*	
**BN1106_s2242B000188**	43	Proteasome subunit beta 2	Proteosome machinery		*	*
**BN1106_s1043B000210**	146	ubiquitin-protein ligase UBR4	Proteosome machinery	*		
**BN1106_s1639B000395**	266	20S proteasome subunit alpha 3	Proteosome machinery		*	*
**BN1106_s325B000622**	498	20S proteasome subunit alpha 3	Proteosome machinery			
**BN1106_s4335B000092**	609	UDP-N-acteylglucosamine pyrophosphorylase 1	Proteosome machinery		*	
**BN1106_s452B000151**	629	UDP-glucose 4-epimerase	Proteosome machinery		*	
**BN1106_s452B000152**	630	UDP-glucose 4-epimerase	Proteosome machinery		*	

**BN1106_s5073B000167**	675	26S proteasome regulatory complex component - Posttranslational modification	Proteosome machinery			*						
**BN1106_s510B000501**	676	26S proteasome non-ATPase regulatory subunit 11	Proteosome machinery			*						
**BN1106_s5276B000036**	690	ubiquitin-activating enzyme E1	Proteosome machinery			*						
**BN1106_s5855B000168**	735	Proteasome beta 1 subunit	Proteosome machinery			*						*
**BN1106_s6576B000103**	774	Ubiquitin	Proteosome machinery		*	*					*	*
**BN1106_s6840B000044**	784	Polyubiquitin-A	Proteosome machinery	*	*	*						
**BN1106_s6922B000040**	788	Proteasome subunit beta type	Proteosome machinery			*						
**BN1106_s79B000381**	840	Ubiquitin carboxyl-terminal hydrolase	Proteosome machinery			*						

**BN1106_s945B000221**	895	ubiquitin-related modifier	Proteosome machinery	*
**BN1106_s1026B000539**	3	Ribosomal protein S10	Protein synthesis machinery	*
**BN1106_s1079B000440**	8	Eukaryotic translation initiation factor 5A	Protein synthesis machinery	*
**BN1106_s11983B000006**	15	Ribosomal protein S14	Protein synthesis machinery	*
**BN1106_s3754B000092**	77	Large subunit ribosomal protein L21e	Protein synthesis machinery	*
**BN1106_s11425B000010**	177	60S ribosomal protein L3	Protein synthesis machinery	*
**BN1106_s116B000323**	182	40S ribosomal protein S12	Protein synthesis machinery	*
**BN1106_s1164B000121**	184	Elongation factor 1-gamma	Protein synthesis machinery	*
**BN1106_s12813B000012**	209	Small subunit ribosomal protein S6e	Protein synthesis machinery	*

**BN1106_s1385 B000133**	223	Small subunit ribosomal protein S18e	Protein synthesis machinery		*
**BN1106_s1628 B000104**	264	60s ribosomal protein L27e	Protein synthesis machinery		*
**BN1106_s1661 B000090**	270	ribosomal protein S2	Protein synthesis machinery		*
**BN1106_s1739 B000159**	282	elongation factor 2	Protein synthesis machinery		*
**BN1106_s1888 B000145**	309	Ribosomal protein L19	Protein synthesis machinery		*
**BN1106_s1935 B000255**	316	60S ribosomal protein L8	Protein synthesis machinery		*
**BN1106_s2104 B000158**	343	60S ribosomal protein L18	Protein synthesis machinery		*
**BN1106_s2398 B000171**	390	40S ribosomal protein S21	Protein synthesis machinery		*
**BN1106_s2474 B000182**	401	ATP-dependent RNA helicase DDX23	Protein synthesis machinery	*	

**BN1106_s2662B000418**	419	60S ribosomal protein L18a	Protein synthesis machinery	*
**BN1106_s2741B000351**	428	ribosomal protein L24e Large subunit	Protein synthesis machinery	*
**BN1106_s2798B000065**	435	Putative 60S ribosomal protein L23a	Protein synthesis machinery	*
**BN1106_s285B000836**	441	Ribosomal protein l7ae	Protein synthesis machinery	*
**BN1106_s3036B000185**	469	Asparagine--tRNA ligase	Protein synthesis machinery	*
**BN1106_s338B000296**	512	Ribosomal protein L26	Protein synthesis machinery	*
**BN1106_s346B000282**	521	Ribosomal protein L11	Protein synthesis machinery	*
**BN1106_s3540B000083**	528	Ribosomal protein	Protein synthesis machinery	*
**BN1106_s3580B000148**	532	ATP-dependent RNA helicase DDX5/DBP2	Protein synthesis machinery	*

**BN1106_s3607B000071**	538	40S ribosomal protein SA	Protein synthesis machinery	*	
**BN1106_s401B000237**	573	40s ribosomal protein S5	Protein synthesis machinery	*	
**BN1106_s410B000447**	584	Large subunit ribosomal protein L14e	Protein synthesis machinery	*	
**BN1106_s4248B000039**	597	Ribosomal protein L7a	Protein synthesis machinery	*	
**BN1106_s4252B000085**	599	elongation factor 1-alpha	Protein synthesis machinery	*	gi|46410394
**BN1106_s446B000198**	621	40S ribosomal protein S8	Protein synthesis machinery	*	
**BN1106_s4543B000079**	631	Elongation factor 1-beta	Protein synthesis machinery	*	
**BN1106_s48B000386**	653	Small subunit ribosomal protein S27e	Protein synthesis machinery	*	
**BN1106_s498B000146**	663	60S ribosomal protein L13	Protein synthesis machinery	*	

**BN1106_s507B000153**	674	40S ribosomal protein S4	Protein synthesis machinery	*
**BN1106_s5230B000038**	687	60S ribosomal protein L12	Protein synthesis machinery	*
**BN1106_s554B000504**	707	Small subunit ribosomal protein S30e	Protein synthesis machinery	*
**BN1106_s5854B000082**	734	60S ribosomal protein L4	Protein synthesis machinery	*
**BN1106_s6136B000050**	754	Ribosomal protein	Protein synthesis machinery	*
**BN1106_s6277B000067**	759	Large subunit ribosomal protein L6e	Protein synthesis machinery	*
**BN1106_s714B000190**	796	Ribosomal protein L30	Protein synthesis machinery	*
**BN1106_s725B000470**	797	Eukaryotic translation initiation factor 3	Protein synthesis machinery	*
**BN1106_s73B000493**	801	60s ribosomal protein L13a	Protein synthesis machinery	*
**BN1106_s801B000129**	844	60S ribosomal protein L5	Protein synthesis	*

			machinery							
**BN1106_s886B000188**	865	Elongation factor 1-beta	Protein synthesis machinery			*				
**BN1106_s914B000124**	873	40S ribosomal protein S20	Protein synthesis machinery			*				
**BN1106_s9429B000021**	893	Large subunit ribosomal protein LP1	Protein synthesis machinery			*				
**BN1106_s5825B000038**	731	lipocalin	s/lipocalin		*					
**BN1106_s10332B000010**	6	Cathepsin L1-like	Secreted proteinase		*					
**BN1106_s13444B000002**	20	Cathepsin B-like	Secreted proteinase		*				*	*
**BN1106_s1861B000097**	32	Legumain-2	Secreted proteinase		*		gi|40643267		*	*
**BN1106_s3518B000132**	71	Lysosomal Pro-X carboxypeptidase - s28	Secreted proteinase		*				*	*
**BN1106_s468B000343**	88	Xaa-Pro dipeptidase-M24	Secreted proteinase		*			FhB02186		
**BN1106_s5163B000012**	95	Cathepsin B-like	Secreted proteinase	*	*	*				
**BN1106_s5602B000082**	101	Cathepsin L-like	Secreted proteinase	*	*					
**BN1106_s6570B000050**	109	Cathepsin B1-like	Secreted proteinase	*		*	gi|27526823			
**BN1106_s6570B000051**	110	Cathepsin B3-like	Secreted proteinase	*	*	*	gi|107921798			

**BN1106_s7079 B000034**	113	Leucine aminopeptidase 2 - M17	Secreted proteinase			*				*
**BN1106_s8177 B000010**	122	Cathepsin L-like	Secreted proteinase							
**BN1106_s8490 B000026**	124	Cathepsin L1-like	Secreted proteinase		*			FhB03790/ FhB03882/ FhB03882/ FhB03693/ FhC11819	*	*
**BN1106_s10139 B000014**	133	Cathepsin L-like	Secreted proteinase	*		*	A5Z1V3			
**BN1106_s10332 B000011**	142	Cathepsin L1-like	Secreted proteinase		*					*
**BN1106_s10947 B000008**	159	Legumain-1	Secreted proteinase							
**BN1106_s1241 B000264**	199	serine carboxypeptidase A - S10	Secreted proteinase		*			FhB02190	*	*
**BN1106_s1252 B000363**	204	Leishmanolysin-like peptidase - M8	Secreted proteinase							
**BN1106_s13034 B000002**	215	Dipeptidyl-peptidase III - M49	Secreted proteinase			*			*	
**BN1106_s1407 B000292**	230	Prolyl oligopeptidase - S9	Secreted proteinase			*				
**BN1106_s14108 B000004**	231	Cathepsin B-like	Secreted proteinase	*		*				
**BN1106_s1620 B000120**	262	Lysosomal Pro-X carboxypeptidase - s28	Secreted proteinase		*			FhB00297	*	*
**BN1106_s16222 B000004**	263	Legumain-1	Secreted proteinase			*				

**BN1106_s1772 B000188**	290	Cathepsin B-like	Secreted proteinase		*							*
**BN1106_s1840 B000150**	301	Cathepsin B4-like	Secreted proteinase	*	*	*						
**BN1106_s19975 B000004**	325	Cathepsin L3-like	Secreted proteinase	*								
**BN1106_s2087 B000065**	337	Legumain-1	Secreted proteinase	*	*	*	Fhep29h09.q1k					
**BN1106_s2303 B000143**	378	Cathepsin L6-like	Secreted proteinase	*		*						
**BN1106_s2684 B000094**	421	calpain	Secreted proteinase			*						
**BN1106_s2882 B000074**	447	Legumain-1	Secreted proteinase									
**BN1106_s2882 B000077**	448	Legumain-1	Secreted proteinase									
**BN1106_s3008 B000074**	464	Cathepsin L3-like	Secreted proteinase	*	*	*	Q9GRW4/Q9GRW6/Q95VA7	A5X483	gi|161347489/gi|222820543/gi|10798509			
**BN1106_s3536 B000078**	527	Cathepsin L-like	Secreted proteinase		*						*	
**BN1106_s373B000290**	547	Cathepsin B-like	Secreted proteinase	*	*	*						
**BN1106_s4000 B000155**	572	Cathepsin L-like	Secreted proteinase		*							
**BN1106_s4187 B000060**	591	Cathepsin L3-like	Secreted proteinase	*	*	*						
**BN1106_s4187 B000061**	592	Cathepsin L-like	Secreted proteinase	*		*						
**BN1106_s4223 B000091**	594	Legumain 3	Secreted proteinase	*	*					FhB00106	*	
**BN1106_s4276 B000065**	603	Metalloprotease ARX1 - without EC number associated	Secreted proteinase			*						
**BN1106_s4482 B000044**	624	Cathepsin B2-like	Secreted proteinase	*		*						

**BN1106_s4511 B000075**	627	cytosol alanyl aminopeptidase - M1	Secreted proteinase			*					
**BN1106_s4636 B000039**	636	Cathepsin L-like	Secreted proteinase		*		A8W7J0				
**BN1106_s5100 B000033**	677	Cathepsin B-like	Secreted proteinase	*	*	*					
**BN1106_s5602 B000083**	711	Cathepsin L4-like	Secreted proteinase	*		*					
**BN1106_s5701 B000008**	722	cathepsin L-like	Secreted proteinase			*					
**BN1106_s5702 B000055**	723	Cathepsin L-like	Secreted proteinase		*						
**BN1106_s5880 B000098**	738	Mastin - S1	Secreted proteinase			*					
**BN1106_s617B 000566**	755	Leucine amino peptidase 1 fragment C-terminal - M17	Secreted proteinase	*	*	*					*
**BN1106_s617B 000567**	756	Leucine amino peptidase 1 fragment N-terminal - M17	Secreted proteinase			*					
**BN1106_s6354 B000017**	762	Cathepsin L1-like	Secreted proteinase		*				FhB03910		
**BN1106_s6635 B000017**	775	Legumain-1	Secreted proteinase	*	*	*					
**BN1106_s6995 B000048**	790	Cathepsin L4-like	Secreted proteinase	*	*	*					
**BN1106_s6995 B000049**	791	Cathepsin L-like	Secreted proteinase	*	*	*					
**BN1106_s7289 B000014**	800	Cathepsin L1-like	Secreted proteinase		*			Q7JNQ9/ Q9NB30/ Q9GRW	FhB03688	*	

								5/Q24940/Q24945/Q6R018				
**BN1106_s7456B000012**	818	Cathepsin L1-like	Secreted proteinase		*							
**BN1106_s7612B000030**	824	Legumain	Secreted proteinase	*	*	*	P80527.1					
**BN1106_s7612B000031**	825	Legumain	Secreted proteinase	*		*	Fhep29h09.q1k					
**BN1106_s7612B000032**	826	Legumain	Secreted proteinase			*						
**BN1106_s793B000177**	841	Cathepsin B-like	Secreted proteinase		*						*	*
**BN1106_s8098B000020**	846	Cathepsin L2-like	Secreted proteinase	*	*						*	
**BN1106_s8462B000006**	855	Cathepsin B4 -like	Secreted proteinase	*	*	*						*
**BN1106_s8881B000009**	866	Cathepsin L-like	Secreted proteinase	*	*							
**BN1106_s9069B000006**	870	legumain-1	Secreted proteinase	*	*	*						
**BN1106_s9304B000006**	887	Cathepsin B-like	Secreted proteinase		*		HAN4015b05.p1kT7			FhB03844		
**BN1106_s98B000759**	902	cytosol alanyl aminopeptidase - M1	Secreted proteinase			*						
**BN1106_s9961B000006**	907	Puromycin-sensitive aminopeptidase	Secreted proteinase			*						
**BN1106_s3864B000104**	79	Serpin	Secreted proteinase inhibitor		*	*	Fhep20a08.q1k					
**BN1106_s122B000261**	195	Serpin B6	Secreted proteinase inhibitor		*	*					*	

**BN1106_s1612B000138**	259	Multi-domain cystatin	Secreted proteinase inhibitor		*	*			*
**BN1106_s1727B000096**	279	Serpin	Secreted proteinase inhibitor		*	*			
**BN1106_s2757B000215**	431	Cystatin	Secreted proteinase inhibitor		*	*			
**BN1106_s318B000274**	487	kunitz-type proteinase inhibitor (Fh-KTM)	Secreted proteinase inhibitor	*	*	*	FhC02704	*	
**BN1106_s3226B000049**	493	serpin	Secreted proteinase inhibitor		*	*			
**BN1106_s3911B000104**	557	kunitz-type proteinase inhibitor (Fh-KTM2)	Secreted proteinase inhibitor						
**BN1106_s4618B000050**	634	Serpin B	Secreted proteinase inhibitor		*	*			
**BN1106_s4651B000094**	638	Stefin-1	Secreted proteinase inhibitor	*	*	*		*	
**BN1106_s5476B000058**	700	inhibidor proteases	Secreted proteinase inhibitor	*					
**BN1106_s8826B000029**	864	kunitz-type proteinase inhibitor	Secreted proteinase inhibitor	*	*		FhC02704		
**BN1106_s1026B000543**	4	Universal stress protein UspA	Signal transducti on			*			

**BN1106_s1551 B000464**	27	KH domain-containing, RNA-binding, signal transduction-associated protein 1	Signal transduction		*			
**BN1106_s1971 B000297**	37	Protein DJ-1	Signal transduction	*	*			
**BN1106_s2316 B000078**	46	cAMP-dependent protein kinase type II-alpha regulatory subunit	Signal transduction		*			
**BN1106_s2848 B000228**	56	SLIT and NTRK-like protein	Signal transduction		*		*	
**BN1106_s3261 B000048**	68	otoferlin a	Signal transduction	*				*
**BN1106_s366B 000435**	74	calumenin-B	Signal transduction		*			
**BN1106_s3904 B000042**	81	14-3-3 protein	Signal transduction	*	*	gi|58263527	*	*
**BN1106_s397B 000170**	83	Tensin	Signal transduction		*			
**BN1106_s4074 B000042**	84	14-3-3 protein	Signal transduction		*			
**BN1106_s4999 B000040**	92	Integrin-linked	Signal transduction		*			

		protein kinase		
**BN1106_s5073B000165**	93	major vault protein	Signal transduction	*
**BN1106_s538B000488**	98	Galectin domain protein	Signal transduction	*
**BN1106_s596B000423**	105	Universal stress protein UspA	Signal transduction	*
**BN1106_s828B000244**	123	Sarcoplasmic calcium-binding protein	Signal transduction	*
**BN1106_s101B000544**	132	Dickkopf-related protein 3	Signal transduction	*
**BN1106_s1139B000359**	170	cell division cycle and apoptosis regulator protein 1-like	Signal transduction	*
**BN1106_s11465B000018**	178	Ras-related protein Rab-14	Signal transduction	*
**BN1106_s1277B000102**	208	Calcium binding protein	Signal transduction	*
**BN1106_s1419B000169**	232	Serine/threonine-protein phosphatase	Signal transduction	*
**BN1106_s1441B000250**	233	Guanine nucleotide-binding protein	Signal transduction	*

		subunit beta-1 (G-prot)							
**BN1106_s1506B000116**	243	Guanine nucleotide-binding protein	Signal transduction			*			
**BN1106_s1560B000153**	250	Calmodulin-like protein 3 (CaM3)	Signal transduction			*			
**BN1106_s1657B000161**	269	Tetraspanin-CD63 receptor	Signal transduction	*	*	*	FhB00703		*
**BN1106_s168B000273**	272	Rab11 family-interacting protein	Signal transduction			*			
**BN1106_s17035B000006**	276	Translationally controlled tumor protein	Signal transduction		*	*			
**BN1106_s1806B000287**	295	Calcium-binding protein	Signal transduction			*			
**BN1106_s1806B000294**	296	Calcium binding protein	Signal transduction		*	*	FhB02185		
**BN1106_s1819B000120**	297	serine/threonine protein kinase	Signal transduction		*				
**BN1106_s1823B000148**	298	Lysosome-associated membrane glycoprotein	Signal transduction		*	*		*	
**BN1106_s1855B000093**	305	adenylate kinase	Signal transduction			*			

**BN1106_s1908 B000177**	313	RAS-like GTP-binding protein	Signal transduction	*	*			*
**BN1106_s204B 000249**	332	Calpain-B	Signal transduction	*	*			
**BN1106_s2053 B000154**	335	mucin	Signal transduction		*			
**BN1106_s2096 B000232**	339	14-3-3 family	Signal transduction		*			
**BN1106_s210B 000998**	340	Calmodulin-like protein 1 (CaM1)	Signal transduction	*	*			
**BN1106_s2124 B000372**	345	Ras-related protein Ral-A	Signal transduction	*				
**BN1106_s214B 000741**	349	Calcium-binding protein	Signal transduction		*	gi|2764758		
**BN1106_s214B 000742**	350	Calcium-binding protein	Signal transduction	*	*		FhB01383	
**BN1106_s214B 000744**	352	CaBP4 (calcium-binding EF-hand protein 4)	Signal transduction		*			
**BN1106_s214B 000747**	354	CaBP3 (calcium-binding EF-hand protein 3)	Signal transduction		*			
**BN1106_s2140 B000163**	356	Calponin	Signal transduction		*			

**BN1106_s2277 B000048**	373	Calmodulin-like protein 2 (CaM2)	Signal transduction	*	*	FhB00790	
**BN1106_s2409 B000122**	392	Calbindin-32	Signal transduction		*		
**BN1106_s2438 B000175**	398	guanine nucleotide-binding protein subunit beta-2-like	Signal transduction		*		
**BN1106_s2487 B000187**	402	Protein NDRG1	Signal transduction		*		
**BN1106_s258B 000276**	411	ras-related protein Rab-8A,	Signal transduction	*			*
**BN1106_s2615 B000090**	415	Myoferlin or Dysferlin	Signal transduction	*	*		
**BN1106_s2673 B000071**	420	Calreticulin	Signal transduction		*		
**BN1106_s2716 B000103**	424	dehydrogenase/reductase SDR family member 1-li	Signal transduction		*		
**BN1106_s280B 000159**	436	Calcium-binding protein	Signal transduction		*		
**BN1106_s2898 B000145**	450	Cystathionine beta-synthase	Signal transduction		*		
**BN1106_s3172 B000053**	477	14-3-3	Signal transduction	*	*		

**BN1106_s318B000272**	480	Calcium-binding EF-hand	Signal transduction			*	
**BN1106_s344B000191**	519	Nucleoside diphosphate kinase - functions of the NDP kinases in the processes of signal transduction in various organisms	Signal transduction		*	*	
**BN1106_s3585B000136**	533	Myoferlin or Dysferlin	Signal transduction		*	*	*
**BN1106_s3590B000078**	534	FERM domain-containing protein	Signal transduction			*	
**BN1106_s4B000834**	569	calcium-binding protein	Signal transduction	*		*	
**BN1106_s4B000836**	570	calcium-binding protein	Signal transduction		*	*	
**BN1106_s417B000229**	590	cAMP-dependent protein kinase type II regulatory subunit	Signal transduction			*	
**BN1106_s4290B000110**	604	Adenylyl cyclase-associated protein 1	Signal transduction			*	

**BN1106_s4512B000085**	628	ADP-ribosylation factor	Signal transduction		*		
**BN1106_s4560B000072**	633	Tetraspanin	Signal transduction	*		*	*
**BN1106_s4672B000098**	641	Rho GDP-dissociation inhibitor	Signal transduction	*	*		
**BN1106_s468B000347**	642	SPARC protein	Signal transduction		*		
**BN1106_s483B000264**	657	Receptor expression-enhancing protein	Signal transduction		*		
**BN1106_s4840B000058**	658	Otoferlin	Signal transduction	*		*	
**BN1106_s519B000125**	684	SWI/SNF-related matrix-associated actin-dependent regulator of chromatin subfamily A-like protein 1	Signal transduction		*		
**BN1106_s605B000204**	749	Rab GDP dissociation inhibitor alpha	Signal transduction		*		
**BN1106_s6059B000016**	750	Calcium-binding protein	Signal transduction	*			

**BN1106_s652B000433**	768	Zinc binding protein	Signal transduction		*			
**BN1106_s678B000118**	780	Calmodulin-like protein 4 (CaM4)	Signal transduction		*	*		*
**BN1106_s686B000273**	785	14-3-3 protein gamma	Signal transduction			*	FhB00240	
**BN1106_s7273B000042**	799	Major vault protein	Signal transduction	*		*		*
**BN1106_s736B000233**	804	Dihydropyrimidinase-like 2	Signal transduction			*		
**BN1106_s773B000382**	832	Calponin	Signal transduction			*		
**BN1106_s7814B000036**	835	Integrin-linked protein kinase	Signal transduction			*		
**BN1106_s844B000259**	853	Rab-protein 11	Signal transduction		*	*		*
**BN1106_s8501B000034**	857	Ras-related protein Rab-5B	Signal transduction			*		
**BN1106_s915B000136**	874	Tetraspanin-1	Signal transduction		*	*		*
**BN1106_s995B000144**	906	protein MEMO1	Signal transduction			*		
**BN1106_s1871B000313**	307	Programmed cell death 6-interacting protein	Signal transduction apoptosis		*		FhC01381	*

**BN1106_s347B000346**	522	programmed cell death-involved protein	Signal transduction apoptosis		*
**BN1106_s3654B000233**	540	programmed cell death protein 6-like isoform X1	Signal transduction apoptosis	*	
**BN1106_s1922B000117.**	34	Small nuclear ribonucleoprotein G	Transcription machinery		*
**BN1106_s2871B000068**	57	poly(A) binding protein	Transcription machinery		*
**BN1106_s2945B000112**	61	seryl-aminoacyl-tRNA synthetase	Transcription machinery		*
**BN1106_s3553B000143**	72	Heterogeneous nuclear ribonucleoproteins A2/B1	Transcription machinery		*
**BN1106_s4644B000085**	87	Heterogeneous nuclear ribonucleoprotein F/H	Transcription machinery		*
**BN1106_s1270B000089**	207	Heterogeneous nuclear ribonucleoprotein	Transcription machinery		*
**BN1106_s135B000521**	220	Template-activating factor I	Transcription machinery		*

**BN1106_s1521B000163**	246	Tar DNA-binding protein	Transcription machinery	*
**BN1106_s1560B000152**	249	RNA-binding region RNP-1 (RNA recognition motif)	Transcription machinery	*
**BN1106_s1575B000264**	251	Transcriptional coactivator	Transcription machinery	*
**BN1106_s1714B000259**	278	SAFB Scaffold attachment factor B2	Transcription machinery	*
**BN1106_s1945B000138**	317	Splicing factor, arginine/serine-rich 7	Transcription machinery	*
**BN1106_s1960B000081**	320	RNA-binding protein 4.1	Transcription machinery	*
**BN1106_s2169B000110**	361	Splicing factor arginine/serine-rich 1/9	Transcription machinery	*
**BN1106_s2968B000206**	459	serine/arginine-rich splicing factor 7-like	Transcription machinery	*
**BN1106_s303B000438**	467	Regulator of differentiation 1	Transcription machinery	*

**BN1106_s3074B000155**	473	transformer-2 protein homolog beta-like	Transcription machinery		*
**BN1106_s310B000140**	474	Heterogeneous nuclear ribonucleoprotein U-like	Transcription machinery		*
**BN1106_s3171B000156**	476	Heterogeneous nuclear ribonucleoprotein K	Transcription machinery		*
**BN1106_s3948B000057**	562	splicing regulatory protein (FUSE binding protein 2)	Transcription machinery		*
**BN1106_s397B000172**	566	Heterogeneous nuclear ribonucleoprotein K	Transcription machinery		*
**BN1106_s5603B000136**	712	Activated RNA polymerase II transcriptional coactivator p15	Transcription machinery		*
**BN1106_s607B000421**	752	Transcription elongation factor B polypeptide 1	Transcription machinery	*	*
**BN1106_s698B000162**	789	small nuclear ribonucleoprotein D1	Transcription machinery		*

**BN1106_s947B000845**	896	RNA-binding protein	Transcription machinery			*		
**BN1106_s986B000164**	903	RNA polymerase II transcription elongation factor,	Transcription machinery		*	*		
**BN1106_s1437B000141**	21	vacuolar protein sorting 4b	Transport/Storage		*			*
**BN1106_s1966B000132**	36	Calponin/Transgelin	Transport/Storage			*		
**BN1106_s2298B000195**	44	Vesicle-associated membrane protein-associated protein A	Transport/Storage			*		
**BN1106_s2471B000098**	53	ATP binding cassette	Transport/Storage		*		*	*
**BN1106_s274B000296**	55	ATP-binding cassette	Transport/Storage		*		*	*
**BN1106_s584B000346**	102	Glucose transporter-2 protein	Transport/Storage	*	*	*		
**BN1106_s584B000348**	103	Solute carrier family 2 facilitated glucose transporter member 3	Transport/Storage			*		

**BN1106_s1061B000222**	150	Alpha/beta hydrolase domain-containing protein	Transport /Storage			*		
**BN1106_s1110B000106**	165	cubilin-like	Transport /Storage	*	*	*	*	*
**BN1106_s114B000614**	171	Cubilin	Transport /Storage	*		*		
**BN1106_s114B000615**	172	Cubilin-like	Transport /Storage	*	*	*		*
**BN1106_s1326B000426**	216	retinitis pigmentosa 1-like 1 protein-like - function not known, probably transport	Transport /Storage			*		
**BN1106_s1326B000429**	217	sodium-coupled monocarboxylate transporter 1-like	Transport /Storage		*			
**BN1106_s1581B000120**	253	Anoctamin	Transport /Storage		*			
**BN1106_s1633B000182**	265	H+-ATPase subunit B	Transport /Storage			*		
**BN1106_s168B000275**	273	GPI-anchored surface glycoprotein	Transport /Storage			*		
**BN1106_s168B000276**	274	GPI-anchored surface glycoprotein	Transport /Storage					

**BN1106_s171B 000376**	277	Vesicle-associated membrane protein 7	Transport /Storage	*			
**BN1106_s17622 B000002**	288	cubilin-like isoform 1	Transport /Storage	*		*	
**BN1106_s18772 B000008**	308	Vacuolar H + ATPase 100kD subunit 1	Transport /Storage		*		*
**BN1106_s1902 B000116**	312	DRP1, density-regulated protein	Transport /Storage			*	
**BN1106_s2110 B000156**	344	V-type H+-transporting ATPase subunit A	Transport /Storage			*	
**BN1106_s223B 000273**	367	Calcium-transporting ATPase	Transport /Storage			*	
**BN1106_s2318 B000216**	380	Alpha-tocopherol transfer protein-like	Transport /Storage				
**BN1106_s2431 B000094**	396	DNA damage-inducible protein 1	Transport /Storage			*	
**BN1106_s2566 B000129**	408	vacuolar protein sorting 26 or von Willebrand factor A	Transport /Storage			*	
**BN1106_s2597 B000195**	413	Charged multivesicular body protein 4	Transport /Storage		*		

**BN1106_s2655 B000264**	418	Charged multivesicular body protein	Transport /Storage		*							*
**BN1106_s2858 B000111**	444	Vacuolar protein sorting-associated protein VTA1	Transport /Storage		*							*
**BN1106_s2916 B000225**	455	Transmembrane protein C2orf18	Transport /Storage		*							
**BN1106_s3001 B000131**	461	cubilin-like	Transport /Storage	*	*	*					*	
**BN1106_s3001 B000132**	462	cubilin-like	Transport /Storage	*	*	*	HAN4005a07.q1kT3					*
**BN1106_s3001 B000132**	463	cubilin-like	Transport /Storage	*	*	*						
**BN1106_s3067 B000141**	472	transporter SVOPL	Transport /Storage			*						
**BN1106_s3313 B000078**	504	ATP:ADP antiporter	Transport /Storage			*						
**BN1106_s3321 B000106**	506	ATPase, H+ transporting, lysosomal accessory protein 1	Transport /Storage		*							
**BN1106_s335B 000427**	511	Endophilin-B1	Transport /Storage			*						
**BN1106_s3396 B000086**	515	ATP-binding cassette subfamily D (ALD) - ABC transporter	Transport /Storage		*					FhC06858		

**BN1106_s3396 B000087**	516	ATP-binding cassette subfamily D (ALD) - ABC transporter	Transport /Storage	*		
**BN1106_s3547 B000116**	529	sodium/potassium-transporting ATPase subunit beta-3	Transport /Storage		*	
**BN1106_s3549 B000112**	530	Voltage-dependent anion channel protein 2	Transport /Storage		*	
**BN1106_s3747 B000111**	548	calponin/transgelin	Transport /Storage		*	gi|29841466
**BN1106_s420B 000182**	593	importin-beta 3	Transport /Storage		*	
**BN1106_s4802 B000087**	654	Mitochondrial import receptor subunit TOM34 - cytosolic cochaperone of the Hsp90/Hsp70 protein complex	Transport /Storage		*	
**BN1106_s4862 B000066**	659	V-type proton ATPase 116 kDa subunit a isoform 1	Transport /Storage	*		

**BN1106_s521B 000167**	686	SNaK1	Transport /Storage			*	*
**BN1106_s5340 B000050**	696	GTP-binding nuclear protein Ran	Transport /Storage			*	
**BN1106_s5369 B000081**	698	Transitional endoplasmic reticulum ATPase	Transport /Storage			*	
**BN1106_s577B 000267**	727	Calcium-transporting ATPase	Transport /Storage			*	
**BN1106_s577B 000269**	728	Calcium-transporting ATPase	Transport /Storage			*	
**BN1106_s584B 000350**	733	Glucose transporter-2 protein	Transport /Storage	*	*	*	*
**BN1106_s6006 B000040**	743	cubilin-like	Transport /Storage		*		
**BN1106_s605B 000203**	748	Putative rab GDP-dissociation inhibitor	Transport /Storage		*	*	
**BN1106_s7307 B000022**	802	cubilin	Transport /Storage	*		*	
**BN1106_s795B 000311**	842	Nuclear transport factor-2	Transport /Storage			*	
**BN1106_s823B 000216**	851	CRAL-TRIO domain-containing protein - sec14 cytosolic factor family	Transport /Storage		*		

**BN1106_s871B000120**	862	B-cell receptor-associated protein	Transport /Storage			*	
**BN1106_s912B000169**	871	Charged multivesicular body protein 2a	Transport /Storage		*		
**BN1106_s9797B000034**	901	cubilin-like protein	Transport /Storage	*	*	*	
**BN1106_s2196B000154**	40	hypothetical protein T265_12952, partial	Unknown conserved			*	
**BN1106_s4413B000122**	86	hypothetical protein	Unknown conserved		*		*
**BN1106_s487B000135**	90	uncharacterized protein	Unknown conserved		*	*	
**BN1106_s1251B000326**	202	tr-G7YKJ4-G7YKJ4_CLOSI OS=Clonorchis sinensis GN=CLF_110325 PE=4 SV=1 - probable fragment	Unknown conserved			*	
**BN1106_s1287B000157**	210	Uncharacterized protein	Unknown conserved		*		
**BN1106_s173B000867**	280	Protein FAM79A - uk function	Unknown conserved			*	
**BN1106_s214B000745**	353	Tegument antigen	Unknown conserved			*	

**BN1106_s214B000748**	355	Tegument antigen	Unknown conserved			*		
**BN1106_s37B000343**	544	conserved hypothetical protein	Unknown conserved			*		
**BN1106_s426B000263**	601	Tegumental protein	Unknown conserved			*		
**BN1106_s574B000139**	724	Unknown product	Unknown conserved	*				
**BN1106_s586B000374**	737	Fasciola/Schistosoma cross-reactive protein	Unknown conserved		*	*		
**BN1106_s606B000244**	751	hypothetical protein T265_05756	Unknown conserved		*			
**BN1106_s739B000131**	805	Tegumental protein	Unknown conserved			*		
**BN1106_s739B000132**	806	hypothetical protein T265_08040	Unknown conserved		*	*	FhC01433	*
**BN1106_s789B000472**	838	hypothetical protein T265_14322, partial	Unknown conserved			*		
**BN1106_s789B000473**	839	protein F37C4.5-like - no known function	Unknown conserved			*		
**BN1106_s1074B000200**	153	Uncharacterized protein	Unknown			*		
**BN1106_s1076B000677**	154	Unknown product	Unknown			*		

**BN1106_s1200 B000196**	193	Unknown product	Unknown	*		*	
**BN1106_s1200 B000197**	194	Unknown product	Unknown			*	
**BN1106_s1233 B000314**	197	Unknown product	Unknown			*	
**BN1106_s1349 B000189**	219	Unknown product	Unknown		*		
**BN1106_s136B 000270**	221	Unknown product	Unknown			*	
**BN1106_s13980 B000012**	225	Unknown product	Unknown	*			
**BN1106_s1536 B000294**	247	Unknown product	Unknown			*	
**BN1106_s1579 B000120**	252	Unknown product	Unknown			*	
**BN1106_s18B0 00411**	294	Unknown product	Unknown			*	
**BN1106_s1830 B000391**	299	Unknown product	Unknown			*	
**BN1106_s19966 B000004**	324	Unknown product	Unknown		*		
**BN1106_s216B 000184**	358	Unknown product	Unknown		*		*
**BN1106_s2275 B000114**	371	Uncharacter ized protein	Unknown	*		*	
**BN1106_s263B 000603**	416	uncharacteri zed protein	Unknown			*	
**BN1106_s2751 B000093**	430	Unknown product	Unknown				
**BN1106_s3270 B000103**	500	Unknown product	Unknown			*	
**BN1106_s3381 B000139.**	513	Unknown product	Unknown		*		
**BN1106_s3381 B000140**	514	Unknown product	Unknown				*
**BN1106_s4307 B000027**	606	Unknown product	Unknown				

**BN1106_s440B 000223.**	616	Unknown product	Unknown		*			
**BN1106_s440B 000226.**	617	Unknown product	Unknown		*			*
**BN1106_s4767 B000027**	651	Unknown product	Unknown		*			*
**BN1106_s4767 B000028**	652	Unknown product	Unknown		*			*
**BN1106_s4811 B000069.**	656	Unknown product	Unknown		*			
**BN1106_s5172 B000090.**	680	Unknown product	Unknown	*		*		
**BN1106_s6032 B000034**	747	Unknown product	Unknown			*		
**BN1106_s6800 B000066**	782	Unknown product	Unknown			*		
**BN1106_s6821 B000024**	783	Unknown product	Unknown		*		*	*
**BN1106_s709B 000651**	794	unknown	Unknown		*			
**BN1106_s7443 B000031**	808	Unknown product	Unknown	*	*	*		
**BN1106_s7704 B000009**	827	Unknown product	Unknown		*	*		
**BN1106_s8038 B000016**	845	Unknown product	Unknown	*	*	*	*	*
**BN1106_s8194 B000020**	850	Unknown product	Unknown			*		
**BN1106_s8592 B000014**	858	Unknown product	Unknown		*			
**BN1106_s8629 B000023**	859	Unknown product	Unknown			*		
**BN1106_s928B 000210**	885	Unknown product	Unknown			*		
**BN1106_s986B 000166**	904	Unknown product	Unknown			*		
**BN1106_s999B 000190**	908	Unknown product	Unknown			*		

**BN1106_s1062 B000110**	152	CRIP	Cysteine rich proteins			*						
**BN1106_s10890 B000012**	157	Peptidase inhibitor 16	Cysteine rich proteins	*		*						
**BN1106_s2878 B000056**	446	GLIPR1-like protein 1	Cysteine rich proteins		*							
**BN1106_s4131 B000138**	588	GLIPR1-like protein 1	Cysteine rich proteins		*							
**BN1106_s5591 B000098.**	708	Peptidase inhibitor 16	Cysteine rich proteins	*		*						
**BN1106_s5591 B000098**	709	Peptidase inhibitor 16	Cysteine rich proteins	*								
**BN1106_s9331 B000024**	888	Peptidase inhibitor 16	Cysteine rich proteins	*								
**Not founded in this study or retrieve no hit in the *F. hepatica* genomic database**							FABP3 (Q9U1G6)	FABP1 (Q9UAS2)	Ubiquitin (gi|66818311)	FABP1 (gi|47115698)	LDL receptor (BN1106_s60B000536)	20S proteasome subunit alpha 6 (BN1106_s1579B000119)
							FABP Fh15 (Q7M4G0)	FABP3 (Q9U1G6)	UCE E2 (gi|34719464)	Peptidyl-prolyl cis-trans isomerase (FhB03741)	Beta-galactosidase (BN1106_s5248B000014)	Proteasome subunit beta type-5 (BN1106_s6770B000051)

<table>
<tr><td>Cathepsin L (A8W638)</td><td>FABP Fh15 (Q7M4G0)</td><td>Histone H4 (gi|194772468)</td><td>Thioredoxin peroxidase (FhB00158)</td><td>Acid phosphatase-like protein (BN1106_s1079B000448)</td><td>Otoferlin (BN1106_s92B000560)</td></tr>
<tr><td>Histone H4 (Fhep10a01.q1k)</td><td>Cathepsin L (Q8T5Z9)</td><td>HSP-90 (gi|226479066)</td><td>Retinal dehydrogenase (FhB03623)</td><td>Branched-chain amino acid aminotransferase (BN1106_s676B000138)</td><td>Inositol transporter (BN1106_s3611B000052)</td></tr>
<tr><td>FABP (HAN3004-1f09.q1k)</td><td>Fg SAP-3 (HAN5007d12.q1kaT3)</td><td></td><td>Ferritin (FhB00163)</td><td>Aspartate aminotransferase (BN1106_s4453B000140)</td><td>Beta-1,4-galactosyltransferase 2 (BN1106_s741B000214)</td></tr>
<tr><td>SjCHGC00176 protein (HAN5016d06.q1kT3)</td><td></td><td></td><td>NPC-2 (FhB03724)</td><td>Glutamate dehydrogenase NAD(P)+ (BN1106_s8641B000016)</td><td>ALIX (BN1106_s2963B000136)</td></tr>
<tr><td>peptydil-prolil cis-trans isomerase (HAN5005h03.q1kaT3)</td><td></td><td></td><td>Ubiquitin L40 precursor (FhB01298)</td><td>EH domain-containing protein 1 (BN1106_s2100B000128)</td><td>Proteasome subunit alpha 7 (BN1106_s7720B000057)</td></tr>
</table>

ATP sintase alpha subunit mitochondrial (FhB00596)	Oestrogen-regulated protein EP45 (BN1106_s4565B000032)	Proteasome subunit alpha type (BN1106_s4981B000066)
Serine proteinase inhibitor (FhB01959)		Proteasome subunit alpha type (BN1106_s3658B000104)
Haemoglobin (FhB00323)		Proteasome subunit alpha 6 (BN1106_s1259B000205)
Glutathione dehydrogenase (FhB01082)		20S proteasome subunit beta 2 (BN1106_s2284B000154)
Calponin (FhB01516)		Proteasome subunit alpha type (BN1106_s9050B000016)
GST (FhB00203)		Phospholipid-translocating ATPase IIB (BN1106_s435B000242)
Aminoacylase		Vacuolar assembly protein

(lrc44928)	(BN1106_s2316B000077)
Programmed cell death protein (FhC00663)	Syntenin-1 (BN1106_s4740B000062)
	Charged multivesicular body protein 5 (BN1106_s6543B000070)
	Ras-related protein (BN1106_s637B000246)
	Alpha-galactosidase (BN1106_s1241B000260)
	Uncharacterised (BN1106_s263B000609)
